# A New Prognostic Risk Model Based on PPAR Pathway-Related Genes in Kidney Renal Clear Cell Carcinoma

**DOI:** 10.1155/2020/6937475

**Published:** 2020-09-22

**Authors:** Yingkun Xu, Xiunan Li, Yuqing Han, Zilong Wang, Chenglin Han, Ningke Ruan, Jianyi Li, Xiao Yu, Qinghua Xia, Guangzhen Wu

**Affiliations:** ^1^Department of Urology, Shandong Provincial Hospital, Cheeloo College of Medicine, Shandong University, Jinan, Shandong 250021, China; ^2^Department of Urology, The First Affiliated Hospital of Dalian Medical University, Dalian, Liaoning 116011, China; ^3^Department of Radiology, Shandong Provincial Hospital, Cheeloo College of Medicine, Shandong University, Jinan, Shandong 250021, China; ^4^The Nursing College of Zhengzhou University, Zhengzhou, Henan 450001, China; ^5^Department of Urology, Shandong Provincial Hospital Affiliated to Shandong First Medical University, Jinan, Shandong 250021, China

## Abstract

**Objective:**

This study is aimed at using genes related to the peroxisome proliferator-activated receptor (PPAR) pathway to establish a prognostic risk model in kidney renal clear cell carcinoma (KIRC).

**Methods:**

For this study, we first found the PPAR pathway-related genes on the gene set enrichment analysis (GSEA) website and found the KIRC mRNA expression data and clinical data through TCGA database. Subsequently, we used R language and multiple R language expansion packages to analyze the expression, hazard ratio analysis, and coexpression analysis of PPAR pathway-related genes in KIRC. Afterward, using the Search Tool for the Retrieval of Interacting Genes/Proteins (STRING) website, we established the protein-protein interaction (PPI) network of genes related to the PPAR pathway. After that, we used LASSO regression curve analysis to establish a prognostic survival model in KIRC. Finally, based on the model, we conducted correlation analysis of the clinicopathological characteristics, univariate analysis, and multivariate analysis.

**Results:**

We found that most of the genes related to the PPAR pathway had different degrees of expression differences in KIRC. Among them, the high expression of 27 genes is related to low survival rate of KIRC patients, and the high expression of 13 other genes is related to their high survival rate. Most importantly, we used 13 of these genes successfully to establish a risk model that could accurately predict patients' prognosis. There is a clear correlation between this model and metastasis, tumor, stage, grade, and fustat.

**Conclusions:**

To the best of our knowledge, this is the first study to analyze the entire PPAR pathway in KIRC in detail and successfully establish a risk model for patient prognosis. We believe that our research can provide valuable data for future researchers and clinicians.

## 1. Introduction

Renal malignancies are the twelfth most common tumors occurring worldwide [1]. Renal cell carcinoma (RCC) is the most common primary malignant tumor of the kidney, accounting for 90% to 95% of all cases of renal cancer [2]. Kidney renal clear cell carcinoma (KIRC) is the most common subtype of RCC [3]. Surgery is the primary treatment for early kidney cancer; however, it usually results in unsatisfactory outcomes, because 20% to 50% of the patients will relapse after surgery, and about 30% of the patients, though they miss local recurrence after surgery, end up having distant metastasis [4]. Renal cell carcinoma is highly resistant to radiotherapy and chemotherapy. Immunotherapy is extraordinarily inefficient and has apparent side effects [5, 6]. Molecular-targeted drug therapy is the primary treatment for advanced renal cancer [7]. In recent years, targeted drugs such as sunitinib have shown sound therapeutic effects and have become first-line treatment for patients with advanced kidney cancer. However, a considerable number of patients with kidney cancer show the original primary resistance and secondary resistance. Therefore, there is an urgent need to find new molecular therapeutic targets. Molecules such as VHL and VEGF were discovered as part of this ongoing quest for new targets for cancer therapy. However, more in-depth understanding of cancer shows that the occurrence of the disease is not the result of uncontrolled single or several oncogenes or tumor suppressors. Oncogenesis is the result of a complex mechanism, which may involve typical serial changes in many critical biological pathways, involving groups of highly related molecules [8, 9]. We use this new understanding to explore the potential role played by entire pathways in kidney cancer, to potentially arrive at successful therapeutic modes of action. Such research helps to understand the pathogenesis of renal cancer and provide personalized treatment.

As a biological pathway mediated by specific receptors, the peroxisome proliferator-activated receptor (PPAR) pathway plays a key role in cell differentiation, development, metabolism (sugar, lipid, and protein), and tumorigenesis. KIRC is also known as clear cell renal cell carcinoma (ccRCC) because its cells contain a large amount of deposited lipids and present a special transparent appearance. PPAR is a nuclear hormone receptor activated by fatty acids and their derivatives [10]. Therefore, we have reason to believe that the PPAR pathway plays an important role in the progress of KIRC. PPAR has three subtypes (PPAR*α*, PPAR*β*, and PPAR*γ*), which show different expressions in vertebrates [11]. They are each encoded by a separate gene and combine fatty acids and eicosanoids. PPAR*α* plays a role in clearing circulating lipids or cellular lipids by regulating the expression of genes involved in lipid metabolism in liver and skeletal muscle. PPAR*β* is involved in lipid oxidation and cell proliferation. PPAR*γ* promotes the differentiation of adipocytes, thereby increasing blood glucose uptake. The PPAR pathway is also considered to be a regulatory pathway for various cancers. PPAR*α* is a potential drug target for the treatment of kidney cancer. In renal cancer cell lines, the PPAR*α* antagonist GW6471 can arrest the cell cycle in G0/G1 phase by attenuating cell cycle regulatory proteins, thereby inducing cell apoptosis. GW6471 can also attenuate fatty acid oxidation and oxidative phosphorylation by inhibiting glycolysis, and thereby inhibit the growth of kidney cancer cells. However, PPAR*α* has been controversial in the regulation of cell growth, proliferation, and tumorigenesis [12, 13]. Some studies have shown that in colorectal cancer, PPAR*α* activated by fenofibrate can stall the progress of colorectal cancer by inhibiting the expression of proinflammatory factors and by increasing the antioxidant capacity of cells [14]. Additionally, activated PPAR*α* can play an anti-inflammatory function by reducing the production of cytokines, which may lead to the downregulation of NF-*κ*B and COX-2 [15, 16]. It has been reported that after the retinoic acid/PPAR*α* pathway is disrupted, it will affect oxidative damage and change the expression of tumor suppressors, which may lead to colorectal tumors caused by low folic acid intake [17]. Shaw et al. found that retinoic acid could also bind to PPAR*β* to promote tumor cell growth and inhibit apoptosis [18]. Previous studies have found that some PPAR*γ* agonists can inhibit tumor cell proliferation, induce tumor cell apoptosis, and inhibit tumor angiogenesis. If used in combination with chemotherapeutics, it is also thought to increase the antitumor effect of the latter [19, 20]. However, some studies have found that activation of PPAR*γ* can promote tumor development [21–25]. All this shows that PPARs and the PPAR pathway are closely related to the occurrence and development of tumors and may become a potential target for tumor treatment.

In our study, we conducted an in-depth and detailed analysis of genes related to the PPAR pathway in KIRC. We analyzed the expression of these genes in KIRC, and found that most of them had apparent expression differences. After conducting hazard ratio (HR) analysis, we found that most of them played a role as a promoter or inhibitor in the occurrence and development of KIRC. We then established a prognostic model composed of 13 PPAR pathway-related genes. The ROC curve results show that this model has good prediction accuracy. In the future, we hope that our research can provide accurate data for later researchers, and at the same time, can help doctors make proper clinical diagnosis and treatment decisions for patients.

## 2. Materials and Methods

### 2.1. Data Collection

In May 2020, we obtained mRNA expression data and clinical data set of KIRC through TCGA database. Then we found the PPAR pathway through the GSEA analysis website (https://www.gsea-msigdb.org/gsea/index.jsp) and evaluated the genes in this pathway. The standard name of this path is KEGG_PPAR_signaling_pathway, and the systematic name is M13088.

### 2.2. Generation of Protein-Protein Interaction Networks

The Search Tool for the Retrieval of Interacting Genes/Proteins (STRING) website can be used to predict the functional correlation between different proteins (https://string-db.org/) [26]. The STRING is a continuously updated biological database that contains comprehensive and easily accessible interactive information, some of which are obtained through experiments and others through predictive analysis. In this study, we used the website's online tool to map the protein-protein interaction (PPI) network between molecules related to the PPAR pathway.

### 2.3. The Human Protein Atlas (HPA) Website

This database (http://www.proteinatlas.org/) contains protein distribution information including those of multiple human tissues and organs and provides tissue and cell expression levels of nearly 20,000 human proteins [27, 28]. We used this website to explore the protein expression of CPT2 in normal kidney tissues and kidney cancer tissues.

### 2.4. Renal Cancer Cell Lines and Plasmid Transfection

In this study, renal cancer cell lines 786-O and ACHN were purchased from the Institute of Cell Research, Chinese Academy of Sciences. These two cell lines were cultured in the presence of penicillin and streptomycin at 37°C in an atmosphere containing 5% CO_2_. We harvested 2 × 10^5^ 786-O and ACHN cells during the logarithmic growth period and seeded them into 6-well plates. The plasmid was transfected the next day. Subsequently, Lipofectamine 2000 (Invitrogen) and plasmid fragments were diluted in serum-free medium, and a pipette was used to add 100 *μ*L of the mixture to the 6-well plates. After 6 hours of incubation at 37°C, the medium containing serum was changed to continue the culture until 24 hours. Finally, the cells were digested with trypsin and collected for proliferation experiments.

### 2.5. Cell Counting Kit-8 (CCK-8) Experiment

First, we cultured 1 × 10^3^ 786-O and ACHN cells per well in a 96-well culture plates (4 replicate wells per group). Subsequently, we added 10 *μ*L of the CCK-8 reagent to each well according to the instructions of Cell Counting Kit-8 (Dojindo, Japan) and incubated it in a 37°C incubator for 1-2 hours. Finally, we used a microplate reader to measure the optical density (OD) of each well at 450 nm, record, analyze, and draw the corresponding histogram.

### 2.6. Data Processing and Analysis

In May 2020, we downloaded RNA-seq transcriptome data of KIRC through the R/Bioconductor software package from TCGAbiolinks, which contains 72 normal kidney tissues and 539 tumor tissues. The clinical information of KIRC patients including age, survival status, grade, stage, tumor (T), and metastasis (M) were all downloaded from TCGAbiolinks and analyzed using Perl language and R studio. We then constructed a heat map that reflects the expression of PPAR pathway-related genes in KIRC. The “pheatmap” expansion package was used to draw heat maps, and the “limma” expansion package was used to analyze mRNA differences. We then performed a hazard ratio (HR) analysis of these molecules in KIRC to show the relationship these molecules have with kidney cancer progression. Afterward, we used the “corrplot” expansion package to plot the coexpression relationship among the PPAR pathway-related genes. Then we used the “glmnet” and “survival” extension packages to draw the LASSO regression curve and survival curve. To verify this model's accuracy, we used the “survival ROC” expansion package to bring a five-year and ten-year ROC curve. Subsequently, based on this model, we analyzed the correlation with the pathological characteristics of renal cell carcinoma patients and depicted it in the form of a heat map. The “rms” software package was used to draw the nomogram. Finally, we combined the clinical data of KIRC patients with the model through the “survival” expansion package for univariate and multivariate analysis.

### 2.7. Statistical Analyses

One-way ANOVA was used to compare the expression of PPAR pathway-related genes in tumor and normal tissue samples. The Student's *t*-test was used to compare the expression of PPAR pathway-related genes in the KIRC dataset according to gender, age, stage, tumor (T), and metastasis (M). Node (N) was not included in the study because it was not verified for a large number of samples in TCGA database. The cut-off value of each risk score in the tumor group was determined using the “survminer” expansion package, and the patients were divided into high- and low-risk groups according to the best cut-off threshold value. The R studio package was used for all statistical analyses. *P* < 0.05 was considered statistically significant.

## 3. Results

### 3.1. The Expression of PPAR-Related Genes in KIRC and the Univariate Cox Regression Analysis in KIRC

**Figure 1:**
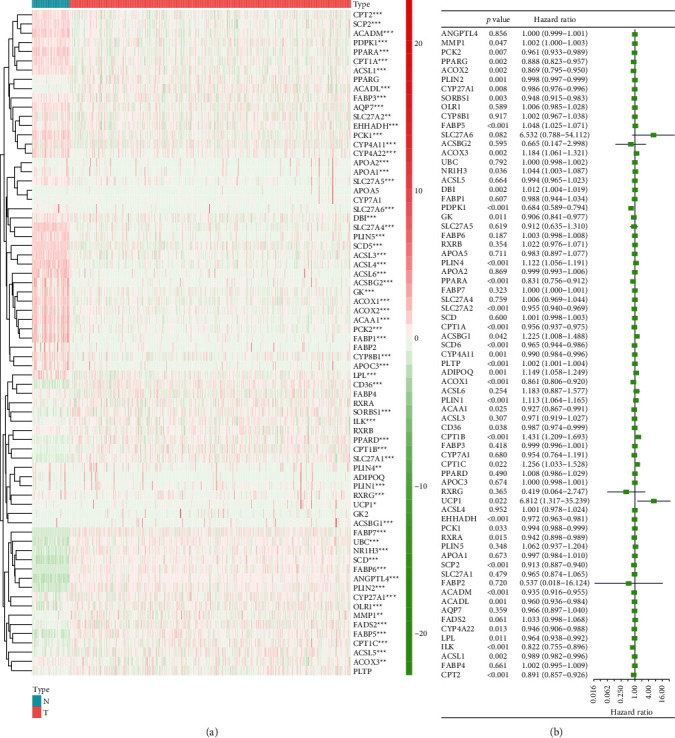
(a) Heat map of PPAR pathway-related gene expression and (b) analysis of their risk factors in KIRC. ^∗^*P* < 0.05; ^∗∗^*P* < 0.01; ^∗∗∗^*P* < 0.001.

To explore the expression of PPAR pathway-related genes in KIRC, we individually plotted their related heat maps (Figure 1(a)). We observed that the vast majority of PPAR pathway-related genes differed significantly in tumor tissues and normal tissues. It can be inferred from this that any change in this pathway plays a significant role in tumorigenesis and progress of the cancer. Then, we performed the univariate Cox regression analysis of these PPAR pathway-related molecules in KIRC (Figure 1(b)). The results show the hazard ratios with 95% confidence intervals (CI) and *P* values for the PPAR pathway-related genes. The results showed that high expression of PCK2, PPARG/PPAR*γ*, ACOX2, PLIN2, CYP27A1, SORBS1, PDPK1, GK, PPARA/PPAR*α*, SLC27A2, CPT1A, SCD5, CYP4A11, ACOX1, ACAA1, CD36, EHHADH, PCK1, RXRA, SCP2, ACADM, ACADL, CYP4A22, LPL, ILK, ACSL1, and CPT2 correlated with better survival rates; in contrast, high expression of MMP1, FABP5, ACOX3, NR1H3, DBI, PLIN4, ACSBG1, PLTP, ADIPOQ, PLIN1, CPT1B, CPT1C, and UCP1 correlated with worse survival rates in KIRC patients.

### 3.2. PPI Network and Coexpression Analysis between PPAR Pathway-Related Molecules

**Figure 2:**
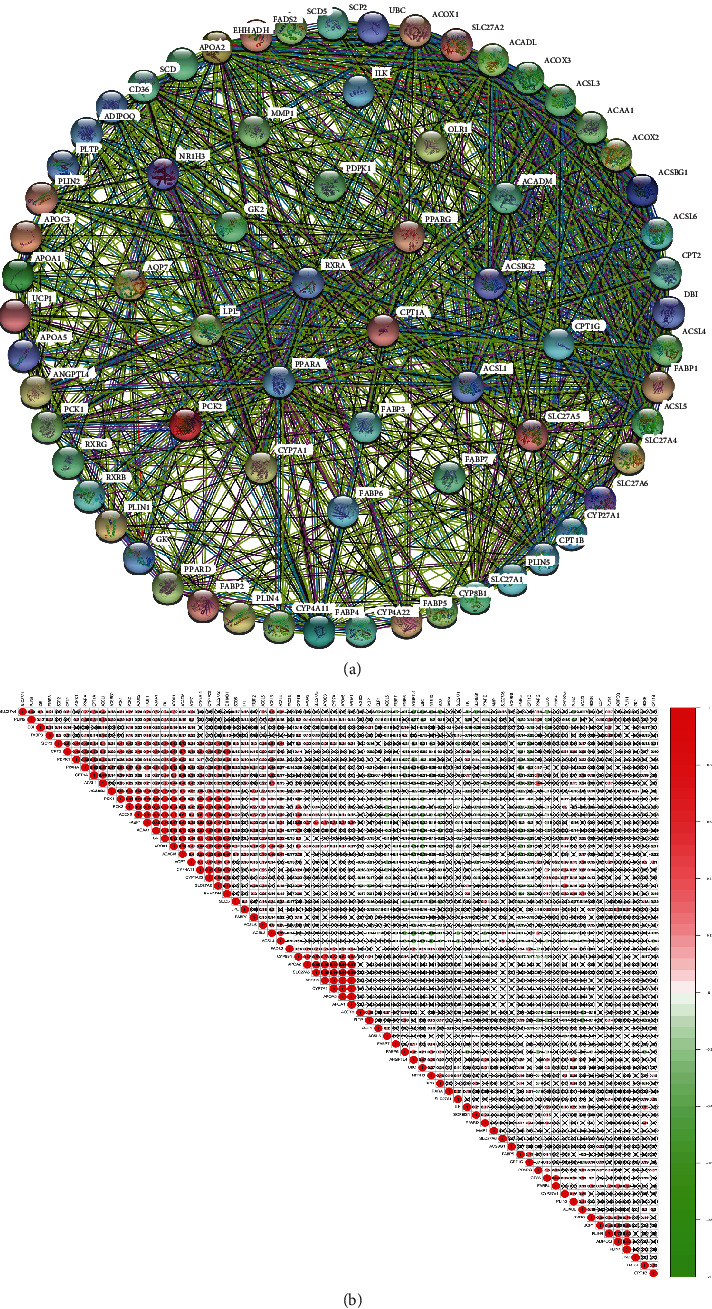
Interactions and correlations between PPAR pathway molecules. PPI network (a) and coexpression analysis (b) between PPAR pathway-related genes.

To explore the interaction between PPAR pathway-related molecules, we used the online tool of the STRING website to illustrate the PPI network (Figure 2(a)). We observed that there were relatively close interactions between the various molecules on the PPAR pathway. When the expansion package on the R language was used to draw an image showing coexpression between molecules (Figure 2(b); Supplementary Table S1 and S2), we observed that there was a highly positive coexpression relationship between the four molecules APOA1, APOA5, APOC3, and CYP7A1. There was a clear positive correlation between PPARA/PPAR*α* and CPT1A, ACSL1, PCK1, PCK2, ACOX2, ACAA1, GK, ACOX1, ACADM, SLC27A2, EHHADH, and so on. Among them, CPT1A was the representative. CPT1A is an important rate-limiting enzyme for fatty acid transport into mitochondria to participate in fatty acid *β*-oxidation, suggesting that PPARA/PPAR*α* may play an important role in lipid metabolism. Additionally, previous studies have shown that CPT1A may be a potential therapeutic target in KIRC [29]. As we all know, KIRC is also known as clear cell renal cell carcinoma because this kidney cancer cell contains many lipid droplets. The presence of apparent abnormalities in fatty acid metabolism in kidney cancer is recognized. In our previous studies, it was found that breaking the lipid homeostasis in this type of kidney cancer could significantly limit tumor progression [30]. Of course, many molecules clearly express correlations, and because of the limited space, they are not listed here.

### 3.3. Using LASSO Regression to Establish a Risk Model Related to the Prognosis of Patients in KIRC

**Figure 3:**
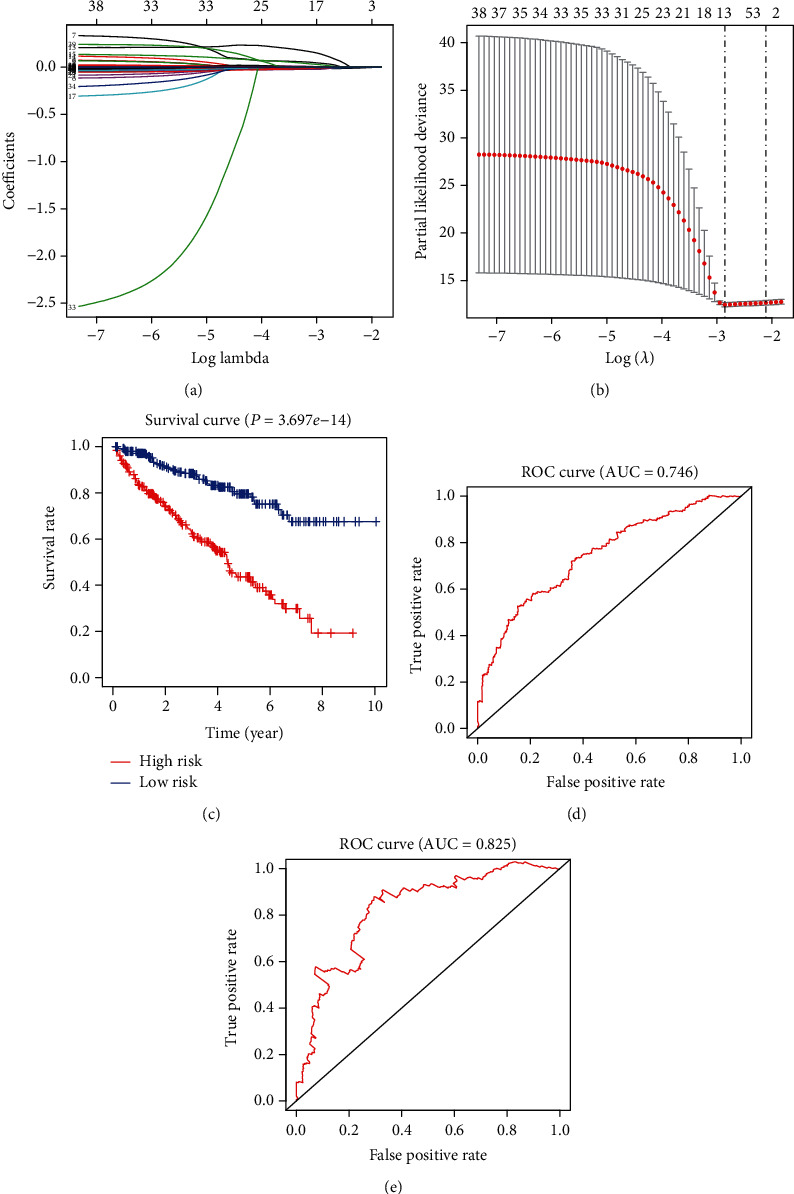
The establishment of the risk model and analysis of its prediction accuracy. (a, b) Target gene selection using LASSO logistic regression. (c) Based on this model, we conducted survival analysis in KIRC. (d) Five-year ROC curve. (e) Ten-year ROC curve.

To explore whether it was possible to use PPAR pathway-related molecules to establish a model in KIRC that could predict the clinical outcome of patients, we conducted a LASSO regression curve analysis (Figures 3(a) and 3(b)). We derived a model composed of thirteen molecules PDPK1, ACADM, SCP2, SLC27A2, EHHADH, CPT2, SCD5, SORBS1, PLTP, FABP5, PLIN1, and PLIN4. We then used this model to divide KIRC patients into a high-risk group and a low-risk group. We observed that the overall survival rate of patients in the high-risk group was significantly lower than that in the low-risk group (*P* = 3.697*e*‐14) (Figure 3(c)). Additionally, we performed ROC curve analysis to analyze the prognostic prediction performance of the new survival model in KIRC patients and obtained a five-year AUC score of 0.746 (Figure 3(d)) and a ten-year AUC score of 0.825 (Figure 3(e)), which indicated that the risk score calculated by the model could accurately predict the 5-year and 10-year survival rates of KIRC patients.

### 3.4. Model-Based Correlation Analysis with Clinicopathological Characteristics, Univariate Cox Regression Analysis, and Multivariate Cox Regression Analysis

**Figure 4:**
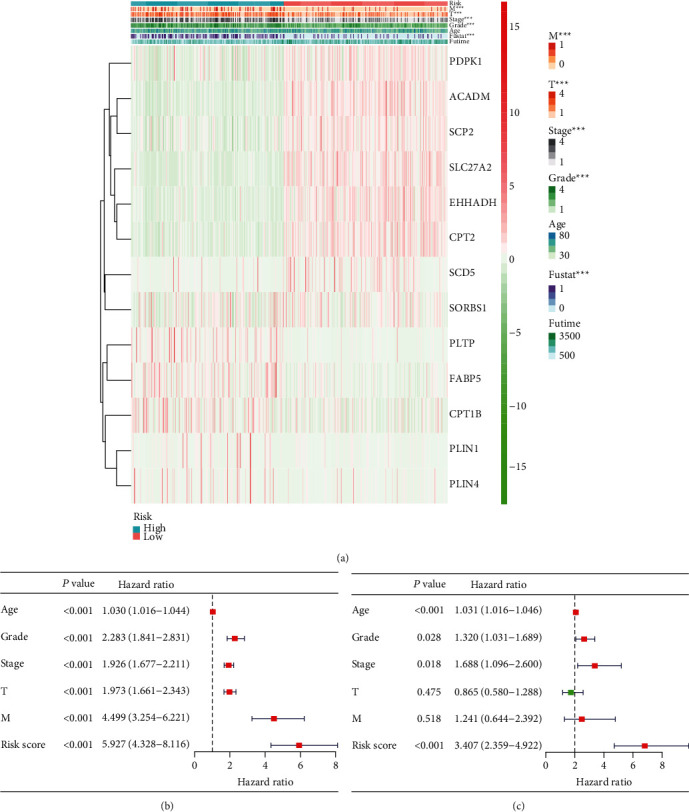
(a) Based on this risk model, the analysis correlates with the clinicopathological characteristics. (b) Univariate Cox regression analysis. The results of univariate Cox regression analysis showed clinicopathological parameters such as age, grade, stage, T (tumor), M (metastasis), and risk score of the new survival model with the OS of KIRC patients. (c) Multivariate Cox regression analysis. The results of multivariate Cox regression analysis showed clinicopathological parameters such as age, grade, stage, and risk score of the new survival model with the OS of KIRC patients. ^∗∗∗^*P* < 0.001.

We plotted a heat map between these target molecules and clinical data to explore the correlation between this risk model and clinicopathological features (Figure 4(a)). In this heat map, we found a relationship between this risk model and the patient's M, T, stage, grade, and fustat. Then we conducted univariate Cox regression analysis (Figure 4(b)) and multivariate Cox regression analysis (Figure 4(c)) and found that this risk model played a risk factor in both different regression analyses. And the hazard ratio of the risk model is higher than that of other clinicopathological features.

### 3.5. Draw a Novel Nomogram Based on Logistic Regression and Verify In Vitro Experiments

**Figure 5:**
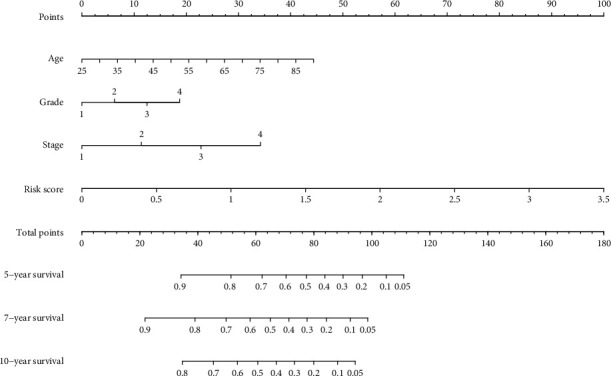
The novel nomogram was constructed based on the risk model for predicting 5-, 7-, or 10-year survival rates in KIRC. The value of each variable gets a score on the dot scale axis. The total score can be easily calculated by adding up each individual score and projecting the total score to a lower total score system, and we can estimate the risk of KIRC patients.

The nomogram predicts the risk of KIRC patients (Figure 5). The value of each variable gets a score on the dot scale axis. The nomogram generates a total of nine rows. The second, third, fourth, and fifth lines represent age, grade, stage, and risk score, respectively. The total score in the sixth row is obtained from the sum of each score assigned to age, grade, stage, and the risk score, and the five-year, seven-year, and ten-year survival rates of KIRC patients can be easily estimated from the total score. Additionally, in order to add validity to our conclusions, we conducted in vitro experiments.

**Figure 6:**
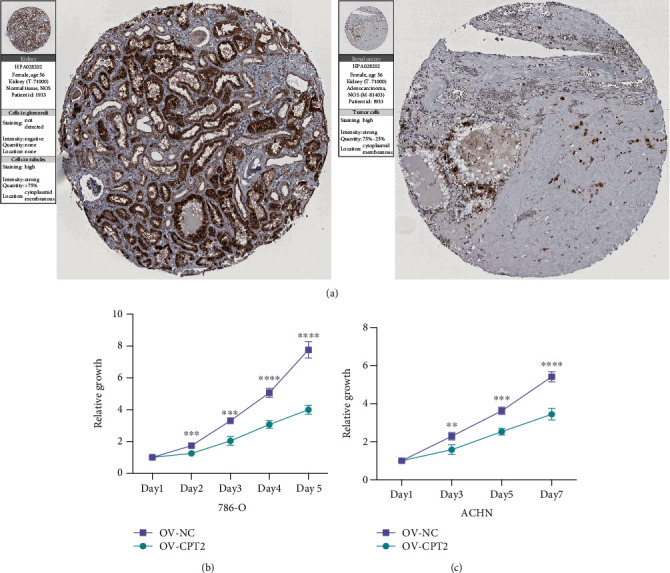
Experimental verification of CPT2. (a) Immunohistochemical images from the HPA database show CPT2 protein expression in normal kidney (N) and KIRC (T) tissues. (b, c) CCK8 assay results show the relative proliferation of OV-NC- and OV-CPT2-transfected 786-O and ACHN cell lines. The data are shown as mean ± S.D. ^∗∗^*P* < 0.01; ^∗∗∗^*P* < 0.001; ^∗∗∗∗^*P* < 0.0001.

By consulting the literature, we found that CPT2 has not been studied in KIRC thus far; hence, we chose CPT2 as a target molecule for subsequent exploration. We explored the expression of CPT2 between normal kidney tissue and renal tumor tissue through the HPA website. We found that the protein expression level of CPT2 in KIRC tissue was significantly lower than that in normal kidney tissue (Figure 6(a)). Subsequently, we used a plasmid transfection technology to establish CPT2 overexpressing renal cancer cell lines (786-O and ACHN cell lines) and conducted CCK-8 experiments. The experimental results showed that the cell proliferation of 786-O and ACHN cells overexpressing CPT2 was significantly inhibited (Figures 6(b) and 6(c)). These results indicate that CPT2 may become a potential therapeutic target in the treatment of renal cancer in the future.

## 4. Discussion

Over the past decade, the research on molecules related to the PPAR pathway in cancer has been ongoing, but the results remain unclear. Some reports suggest that the PPAR pathway plays a protective factor in tumorigenesis, and the primary mechanism is achieved by inhibiting the activity of inflammation or angiogenesis [31, 32]. At the same time, other researchers regard it as a promoter of tumorigenesis [33]. Recently, another study showed that PPARG/PPAR*γ* overexpression might help to better treat patients with CRC by inhibiting the process of EMT (epithelial-mesenchymal transition) [34]. PPARA/PPAR*α* in KIRC can overcome sunitinib resistance by regulating the NF-*κ*B pathway [35]. PPARG/PPAR*γ* in KIRC can promote cell apoptosis and inhibit cell migration and proliferation by inhibiting SIX2 [36, 37]. Similarly, in this investigation, the hazard ratio results show that PPARG/PPAR*γ* plays a protective factor in KIRC. This study aims at integrating PPAR pathway-related genes and determining a model that can predict patients' survival. In our study, we first investigated the expression and prognosis of PPAR pathway-related genes in KIRC. Subsequently, the interaction and coexpression of these molecules were analyzed and through the LASSO regression and cross-validation, the novel thirteen-gene model was determined. This model divides KIRC patients into high-risk and low-risk groups through the risk score, and survival curve analysis shows that the survival of patients in the high-risk group is significantly worse than that of the low-risk group patients. The ROC curve shows that this model has good five-year and ten-year survival prediction accuracy. Multivariate Cox regression analysis shows that this new 13-gene prognostic model is an independent risk factor for KIRC.

The occurrence and development of clear cell renal cell carcinoma is a complex process regulated by genetic changes of multiple molecules. To date, there have been many previous studies exploring the prognostic role of risk models in predicting KIRC patients [38–41]. The clinical application results based on multiple gene expression profiles indicate that genetic risk model may be a promising clinical diagnosis and treatment method [42–44]. In this study, we successfully used 13 genes of PDPK1, ACADM, SCP2, SLC27A2, EHHADH, CPT2, SCD5, SORBS1, PLTP, FABP5, PLIN1, and PLIN4 to construct risk models related to the prognosis of KIRC patients. Its five-year ROC curve has an AUC value of 0.746, and its ten-year ROC curve has an AUC value of 0.825. Generally, a risk model with an AUC value over 0.7 indicates a very high prediction accuracy.

PDPK1 is a regulated protein kinase from the AGC protein kinase family, which can activate multiple downstream effectors associated with various pathways of tumorigenesis [45]. Previous researchers found that inhibiting PDPK1 expression in small cell lung cancer and melanoma can inhibit tumor progression [46, 47]. ACADM can catalyze the first dehydrogenation process of *β*-oxidation of fatty acyl-CoA [48]. In KIRC, the low expression of ACADM may affect the metabolism of medium-chain fatty acids, and then the metabolism of triglycerides, and play an essential role in apoptosis through the function of light chains [49, 50]. Previous studies have shown that SCP2 plays a critical role in stabilizing PML expression. Low expression of SCP2 will increase the phosphorylation level of PMLS518, thereby reducing PML. The downregulation of PML is related to the occurrence and development of high-grade tumors [51]. In endometrial and ovarian cancer, researchers found that SLC27A2 can play a biological role in regulating chemical resistance [52, 53]. EHHADH can encode a bifunctional enzyme and is one of the four enzymes for peroxisomal *β*-oxidation [54]. In the studies of Cablé et al. and Suto et al., it was found that EHHADH had a significantly low expression in colon cancer and hepatocellular carcinoma and could be used as a potential prognostic marker [55, 56]. PPARA/PPAR*α* mainly regulates the expression of CPT2. Studies have shown that in hepatocellular carcinoma, CPT2 is the rate-limiting enzyme for fatty acid oxidation. Its expression level is related to the proliferation and invasion of liver cancer cells. Silencing CPT2 can induce chemical resistance to cisplatin [57]. Some researchers have found that downregulating the expression of CPT2 helps to avoid the lipid toxicity caused by the lipid-rich cell environment [58]. SCD5 is a protein-coding gene that can regulate neuronal cell growth and differentiation by regulating key fat-generating pathways. SCD5 may play an important role through the PPAR pathway in breast cancer chemotherapy [59]. The activity of SCD5 is also correlated with the activity of multiple cancer pathways such as AKT, WNT, and EGFR [60].

SORBS1, also known as Cap/Ponsin protein, can regulate biological processes such as growth factor signaling, cell adhesion, and cancer metastasis [61]. The primary function of the protein encoded by PLTP is to transfer phospholipids from triglyceride-rich lipoproteins to high-density lipoproteins. Studies have shown that PLTP can induce anti-inflammatory responses by activating the ABCA1/STAT3 pathway [62, 63]. FABP5 is a member of the FABP family [64]. Some researchers have found that it can affect the progress of tumors by affecting the activity of the PI3K/AKT signaling pathway in KIRC [65], and that it plays a role as an oncogene in KIRC [66]. CPT1B is one of three CPT1 subtypes. The researchers found that knocking down the expression of CPT1B in BLCA could increase the ability of tumor proliferation and invasion by inhibiting FAO [67]. Overexpression of PLIN1 can inhibit the proliferation, migration, and invasion of breast cancer cells. The expression level of PLIN1 is correlated with the prognosis of breast cancer patients and is expected to become a potential new gene therapy target for breast cancer [68]. PLIN1 may affect tumor progression through PPARG/PPAR*γ* pathway in breast cancer [69]. At the same time, another member of the same family, PLIN4, has also been identified as a therapeutic target for triple-negative breast cancer [70].

It can be inferred from the above-mentioned information that the target gene used to establish this model in this study has received varying degrees of attention and research in various tumors. However, there are parameters like CPT2, SORBS1, PLIN1, and PLIN4 that have not been studied in KIRC. These biomarkers may be worthy of attention in the future of KIRC research. In particular, we explore the potential role of CPT2 in KIRC through in vitro experiments. When we overexpressed CPT2 in 786-O and ACHN kidney cancer cell lines, the cell proliferation ability was significantly inhibited. Hereafter, we will continue to explore the potential role of these genes in KIRC. We believe that this model related to patient prognosis can help doctors choose more personalized treatment for KIRC patients in the future. After the grouping of this model, for patients in the low-risk group, clinicians can appropriately reduce the frequency of examinations and reduce the economic pressure on patients. For patients in the high-risk group, clinicians would be able to give more intensive treatments and strengthen follow-up and regular physical examination to monitor the development of the disease.

## 5. Conclusions

In this study, we used 13 genes related to the PPAR pathway to establish a new prognostic risk model in KIRC, which could accurately predict the five-year and ten-year survival rates of patients. However, it must be admitted that in this study, these thirteen target genes have not been thoroughly explored in KIRC. In the future, large-scale single-center or multicenter clinical validation of this risk model is needed. However, we believe that our research can provide valuable data for future scientific research and clinical practice.

## Data Availability

The data used to support the findings of this study are available from the corresponding author upon request.

## Abbreviations

PPAR: Peroxisome proliferator-activated receptor
KIRC: Kidney renal clear cell carcinoma
GSEA: Gene set enrichment analysis
TCGA: The Cancer Genome Atlas
PPI: Protein-protein interaction
LASSO: Least absolute shrinkage and selection operator
RCC: Renal cell carcinoma
ccRCC: Clear cell renal cell carcinoma
VHL: Von Hippel-Lindau tumor suppressor
VEGF: Vascular endothelial growth factor
PDPK1: 3-Phosphoinositide dependent protein kinase 1
ACADM: Acyl-CoA dehydrogenase medium chain
SCP2: Sterol carrier protein 2
SLC27A2: Solute carrier family 27 member 2
EHHADH: Enoyl-CoA hydratase and 3-hydroxyacyl CoA dehydrogenase
CPT2: Carnitine palmitoyltransferase 2
SCD5: Stearoyl-CoA desaturase 5
SORBS1: Sorbin and SH3 domain containing 1
PLTP: Phospholipid transfer protein
FABP5: Fatty acid binding protein 5
PLIN1: Perilipin 1
PLIN4: Perilipin 4.

## Supplementary Materials

Supplementary materials Table S1: correlation value for coexpression analysis. Table S2: *P* value for coexpression analysis.
