# Supplementary material for: A New Prognostic Risk Model Based on PPAR Pathway-Related Genes in Kidney Renal Clear Cell Carcinoma
Source: PPAR Res. 2020 Sep 22;2020:6937475. doi: 10.1155/2020/6937475 (PMC7527891; doi:10.1155/2020/6937475)
Supplement: Supplementary materials — Table S1: correlation value for coexpression analysis. Table S2: P value for coexpression analysis. [file 6937475.f1.pdf]

Table S1: Correlation value for co-expression analysis

	ANGPTL4	MMP1	PCK2	PPARG	ACOX2	PLIN2
ANGPTL4	1	-0.0184464	-0.2552096	-0.0811196	-0.2565425	0.38591585
MMP1	-0.0184464	1	-0.0288346	-0.0316139	-0.0354995	-0.0071184
PCK2	-0.2552096	-0.0288346	1	-0.0801206	0.90302885	-0.1005927
PPARG	-0.0811196	-0.0316139	-0.0801206	1	-0.0629468	0.02951194
ACOX2	-0.2565425	-0.0354995	0.90302885	-0.0629468	1	-0.094205
PLIN2	0.38591585	-0.0071184	-0.1005927	0.02951194	-0.094205	1
CYP27A1	0.11953494	-0.012831	0.19819217	0.08078134	0.12914273	0.2553952
SORBS1	0.11789041	-0.0267564	-0.1827901	0.0062488	-0.156086	0.04512845
OLR1	-0.0263707	0.03364119	-0.0556009	-0.1482729	-0.0765882	0.12987974
CYP8B1	-0.0655206	-0.0336306	0.45386447	-0.1743476	0.47595452	-0.0768912
FABP5	0.21957362	0.08329121	-0.227727	-0.0966754	-0.2683439	-0.0006737
SLC27A6	-0.1222707	0.08403352	-0.0037225	0.11333393	0.00291751	-0.1134706
ACSBG2	-0.1976122	-0.0342699	0.67848119	-0.0753198	0.64557743	-0.1483702
GK2	0.02048176	-0.000965	-0.0330438	0.04218702	-0.0406508	-0.0302085
ACOX3	0.01573885	0.10236799	-0.0878719	-0.1911023	-0.0735226	-0.0799165
UBC	0.47351863	0.02879384	-0.3326499	-0.0940304	-0.3282984	0.28326051
NR1H3	0.2573668	-0.0161351	-0.1280569	-0.141598	-0.1938236	0.27662187
ACSL5	-0.0236537	-0.0476122	-0.2494405	-0.0716529	-0.250804	0.08403133
DBI	-0.1103342	-0.0179904	0.18980618	-0.0053111	0.22205818	-0.0555311
FABP1	-0.2579715	-0.0261851	0.85480117	-0.0976665	0.81023099	-0.1626149
PDPK1	-0.3961516	-0.0964069	0.24170278	0.08831051	0.29469209	0.0128224
GK	-0.2881527	-0.0465161	0.767012	0.0694413	0.73107896	0.0695314
SLC27A5	-0.0561427	-0.0120759	0.13759091	0.01440373	0.16134863	-0.0755516
FABP6	0.34527758	0.01443056	-0.1628133	-0.2422451	-0.150421	0.11854659
RXRB	-0.0444228	-0.0362883	-0.0200656	-0.0315142	0.03598232	-0.0161433
APOA5	-0.0428588	-0.0039477	0.11322572	-0.0135774	0.14082019	-0.0408087
PLIN4	-0.0843185	-0.0045751	0.03346701	0.15508783	0.08976907	-0.0310618
APOA2	-0.0426137	-0.0052138	0.11168736	-0.0007511	0.14225503	-0.0089136
PPARA	-0.3916332	-0.0849679	0.57326176	0.16937544	0.59576735	-0.0276388
FABP7	0.17461914	-0.0106361	-0.1343003	-0.0508516	-0.1166141	0.04832058
SLC27A4	-0.3749133	-0.0259574	0.25915954	0.18595965	0.24074672	-0.3194843
SLC27A2	-0.1207742	-0.0600194	0.65920175	0.07559305	0.62884401	0.16221768
SCD	0.22003117	0.00911221	-0.2194823	-0.1536681	-0.2495347	0.28359116
CPT1A	-0.2358797	-0.0832411	0.22665495	0.3250108	0.25053133	0.0713875
ACSBG1	-0.0668969	-0.0104311	-0.0241919	0.08818939	-0.0420459	-0.086278
SCD5	-0.268243	-0.0314069	0.18057151	-0.0733852	0.22043423	-0.2740956
CYP4A11	-0.0513418	-0.0389765	0.64246509	0.07066137	0.61015763	0.22861613
PLTP	0.09527797	0.02242799	-0.098961	-0.2578443	-0.0845911	-0.1302079
ADIPOQ	-0.0591563	-0.0071935	-0.0173237	0.01531389	0.00564303	-0.0391242
ACOX1	-0.3617371	-0.076246	0.73917787	0.05958804	0.70378269	0.0153479
ACSL6	-0.2341075	-0.0359063	0.30982154	0.03205997	0.30853388	-0.1248066
PLIN1	0.00186826	-0.0106968	-0.0351737	0.00394715	-0.0060732	0.01790474
ACAA1	-0.2718777	-0.0508101	0.84870732	-0.0333389	0.80130924	-0.1369281
ACSL3	-0.4752871	-0.059093	0.188907	0.25410135	0.18130362	-0.2227233

CD36	0.2119116	-0.0047645	-0.1549219	0.29905613	-0.152621	0.26480918
CPT1B	0.1020781	-0.0203718	-0.1309509	0.17006525	-0.1275456	-0.0084242
FABP3	0.01852679	-0.0510027	0.16273771	0.06178096	0.21334355	0.09786583
CYP7A1	-0.041902	-0.0028154	0.1106209	-0.0121746	0.13918922	-0.0361957
CPT1C	0.09974077	0.00929119	-0.2164113	-0.1390696	-0.2265228	-0.0898239
PPARD	0.28767735	0.02419079	-0.1911449	-0.0511146	-0.1775097	-0.0092928
APOC3	-0.0442978	-0.0042835	0.12118232	-0.0172142	0.14740606	-0.0390897
RXRG	-0.0917775	-0.0362096	0.05522595	0.15462351	0.08710093	0.03305595
UCP1	-0.1031845	-0.0177238	-0.0468862	0.16595761	-0.0259706	-0.0908845
ACSL4	-0.3947396	-0.0049857	0.31699864	-0.0777993	0.32040565	-0.2752336
EHHADH	-0.1050967	-0.0628078	0.58221116	0.02081511	0.57596335	0.34747955
PCK1	-0.2289249	-0.0396967	0.80945237	-0.0271354	0.73631881	-0.0627752
RXRA	0.0481344	-0.0599623	0.10815239	-0.1579045	0.12622491	0.11398718
PLIN5	-0.2749759	-0.0076209	0.20663042	0.25983559	0.23052332	-0.303839
APOA1	-0.0432919	-0.0044053	0.11914094	-0.0145835	0.1465074	-0.0385659
SCP2	-0.3018309	-0.0488665	0.43021477	0.08420648	0.42137216	0.04276251
SLC27A1	0.10686011	-0.0367134	-0.1651765	0.11676555	-0.0786412	0.01068696
FABP2	-0.1556754	-0.0011264	0.27384271	0.02953004	0.31829928	-0.0938445
ACADM	-0.3678096	-0.0673837	0.69224946	0.05023034	0.67474987	-0.0046794
ACADL	0.11968302	-0.0602532	-0.0210207	0.31090983	-0.0155974	0.40947356
AQP7	-0.1511559	-0.0486845	0.424281	0.23069532	0.39712027	0.14655603
FADS2	0.22196418	-0.0125808	-0.1046031	-0.1588155	-0.1068174	0.13959196
CYP4A22	-0.0478059	-0.0347657	0.61074204	0.06959438	0.54206067	0.17625491
LPL	-0.1864044	-0.0249779	0.06533355	0.15301874	0.08174488	-0.1144486
ILK	-0.0561946	-0.0740632	-0.0916335	0.06781931	-0.0512705	0.28144583
ACSL1	-0.2373994	-0.0255984	0.21819622	0.43924522	0.26003481	0.14871317
FABP4	0.07373104	-0.0185605	-0.0184029	0.29179908	-0.0112001	0.11361599
CPT2	-0.1925362	-0.0765418	0.42385972	0.08487787	0.42207226	0.27728603

CYP27A1	SORBS1	OLR1	CYP8B1	FABP5	SLC27A6	ACSBG2
0.11953494	0.11789041	-0.0263707	-0.0655206	0.21957362	-0.1222707	-0.1976122
-0.012831	-0.0267564	0.03364119	-0.0336306	0.08329121	0.08403352	-0.0342699
0.19819217	-0.1827901	-0.0556009	0.45386447	-0.227727	-0.0037225	0.67848119
0.08078134	0.0062488	-0.1482729	-0.1743476	-0.0966754	0.11333393	-0.0753198
0.12914273	-0.156086	-0.0765882	0.47595452	-0.2683439	0.00291751	0.64557743
0.2553952	0.04512845	0.12987974	-0.0768912	-0.0006737	-0.1134706	-0.1483702
1	-0.0732643	0.08601243	0.00372353	-0.0319218	-0.0691589	0.09169451
-0.0732643	1	-0.028286	-0.0230532	-0.016361	-0.0418084	-0.1072776
0.08601243	-0.028286	1	0.03998512	-0.0145654	-0.033823	-0.023355
0.00372353	-0.0230532	0.03998512	1	-0.1704566	-0.0190714	0.3050675
-0.0319218	-0.016361	-0.0145654	-0.1704566	1	-0.0113328	-0.2202652
-0.0691589	-0.0418084	-0.033823	-0.0190714	-0.0113328	1	-0.0216108
0.09169451	-0.1072776	-0.023355	0.3050675	-0.2202652	-0.0216108	1
-0.058529	-0.0117554	-0.0369056	0.00023304	0.00045586	0.02062496	0.16784915
-0.1841377	-0.0524305	0.08696495	0.11289699	0.06137453	-0.0450073	-0.0532691
0.04025506	0.12412495	0.09263961	-0.1266433	0.16037993	-0.0893609	-0.220428
0.30438765	0.07352819	0.15151857	-0.0131389	0.16335788	-0.1090408	-0.0821399
-0.236963	0.13959856	0.20077548	0.06239639	0.00399561	0.04829432	-0.1798038
0.13075637	-0.2542779	0.0498518	0.14959259	0.01518635	0.07295675	0.06047862
0.09977365	-0.168547	-0.0380812	0.55177179	-0.2046714	0.01940778	0.58358604
-0.0586218	0.136992	-0.0010169	0.1300219	-0.3575428	-0.0100158	0.17223613
0.23279365	-0.152004	0.09682226	0.29961575	-0.2639404	-0.0069981	0.5864994
0.06479976	-0.0622331	-0.0725998	0.52848026	-0.0450806	-0.0014273	0.03128378
-0.0819367	-0.0543223	0.05374414	0.16374266	0.09996635	-0.0587584	-0.0626701
0.05802648	-0.03862	-0.1206216	-0.1718209	-0.0471344	-0.0888356	0.04157649
0.0771467	-0.0258044	-0.0377497	0.52373896	-0.0272587	-0.0022158	-0.0205669
-0.0140053	-0.0087114	-0.0364109	0.06036387	-0.0620882	0.11551192	0.05252742
0.09658088	-0.037438	-0.0445905	0.52073377	-0.0158394	-0.0004628	-0.0108518
0.05174028	-0.0105695	-0.0466203	0.26084227	-0.39737	-0.0246174	0.46798953
0.05618786	0.09778553	0.00941359	-0.0461225	0.09561967	-0.042039	-0.0703605
-0.0997663	-0.2937454	-0.2332084	0.08698709	-0.0903474	0.15564377	0.17891976
0.45190615	-0.0885928	-0.0338692	0.25136283	-0.2541938	-0.0661098	0.40667086
0.04220748	0.28905901	0.0845879	-0.0498514	0.15614811	-0.0673619	-0.1278558
0.03740769	0.0305378	-0.1437357	-0.0145987	-0.310168	0.02137435	0.14255246
-0.0311878	0.01157215	-0.0589473	-0.0278481	0.06602863	0.03241509	0.04826549
-0.1185252	-0.0703266	-0.0355825	0.06292317	-0.2355939	-0.0024364	0.14217043
0.33926966	-0.1512548	-0.0445173	0.24408015	-0.1861062	-0.0442577	0.45652874
-0.1443878	-0.0205932	0.07805209	0.20736782	0.1907993	0.03815046	-0.0255541
-0.0237674	0.0666859	-0.0170975	0.00085993	-0.0124747	0.05192187	0.01455881
0.14615864	-0.0905919	-0.0231514	0.4016315	-0.3287432	-0.012371	0.51644211
-0.017595	-0.1349214	-0.098053	0.09496482	-0.1888221	0.07155706	0.29045678
-0.0062888	0.07591686	0.00188443	-0.0105086	-0.0062211	0.04368936	0.05298595
0.12349254	-0.2215697	-0.0430086	0.38542909	-0.281646	0.01525333	0.62027032
-0.2165928	0.00171484	-0.1370874	-0.0166559	-0.1724961	0.20643933	0.15242244

0.18720512	0.15167801	-0.1159945	-0.2289291	0.14342416	-0.0813281	-0.1570208
0.01511576	-0.0472341	0.03156742	-0.1265229	0.06521577	-0.0090893	0.06170643
0.00950942	-0.1545023	0.03403505	0.20470272	-0.136454	0.06086393	0.04307571
0.07923807	-0.0250404	-0.0399192	0.52478	-0.0244848	-0.0022638	-0.0231245
-0.1153263	0.0730931	-0.1928381	-0.1700182	0.40676393	-0.0211661	0.04956101
-0.0007135	0.22521357	0.03566595	-0.139966	0.16892974	-0.0799467	-0.1146425
0.07874436	-0.0263998	-0.0387512	0.5288043	-0.0294788	-0.0023431	-0.0163926
0.09943393	0.08094604	-0.1125661	-0.0340611	-0.1150479	0.02477657	0.13720689
-0.0806215	0.01395011	-0.0613283	-0.0342974	-0.0106936	0.1258256	0.12566021
-0.2253568	-0.023419	-0.010089	0.18904254	-0.1791798	0.25888894	0.17964953
0.32055371	-0.0375902	0.07718155	0.25898407	-0.2970308	-0.0626125	0.38468908
0.16795848	-0.1263646	-0.0715954	0.32216294	-0.1956922	-0.0150902	0.54950283
0.02363279	0.08816373	-0.2193242	0.05053849	0.04358726	-0.0385574	0.02573618
-0.1491374	-0.1044161	-0.1484185	0.20221083	-0.2207458	0.06190982	0.22309543
0.07915658	-0.0288915	-0.037836	0.52801219	-0.0267039	-0.0001732	-0.0176963
0.0641418	0.00628156	-0.0009544	0.3008154	-0.2679475	0.08750672	0.20599089
0.06380785	0.01258192	-0.0519982	-0.1057345	-0.0287029	0.04387513	-0.0986568
0.00830455	0.02249648	0.04405628	0.1786331	-0.1360759	0.01598313	0.32722752
0.07719417	-0.1050449	-0.0218584	0.26440035	-0.3259923	0.00343876	0.50130565
0.38572463	0.08326962	0.05433059	-0.114645	-0.1976467	-0.0414237	0.05156682
0.18351501	-0.2091088	-0.1224662	0.054687	-0.2096215	-0.0241348	0.32316909
0.02165728	0.14628734	-0.0014839	0.15925977	0.13537348	-0.0229897	-0.0162169
0.27441249	-0.1610945	-0.0513565	0.22815961	-0.1508233	-0.0270785	0.41740453
-0.0347746	-0.0528517	-0.182184	-0.1092552	0.00289436	-0.0246191	0.04712412
0.15852175	0.21064747	0.02793373	-0.1352696	-0.0255811	-0.0843932	-0.227708
0.03404751	-0.105966	-0.1313542	0.04257302	-0.2835496	0.04259748	0.07712442
0.12540973	0.00352668	-0.0824305	-0.0857437	0.04435819	-0.0113781	-0.0311434
0.23967214	-0.1300301	0.01911847	0.13835231	-0.3445525	-0.0174047	0.23269867

GK2	ACOX3	UBC	NR1H3	ACSL5	DBI	FABP1
0.02048176	0.01573885	0.47351863	0.2573668	-0.0236537	-0.1103342	-0.2579715
-0.000965	0.10236799	0.02879384	-0.0161351	-0.0476122	-0.0179904	-0.0261851
-0.0330438	-0.0878719	-0.3326499	-0.1280569	-0.2494405	0.18980618	0.85480117
0.04218702	-0.1911023	-0.0940304	-0.141598	-0.0716529	-0.0053111	-0.0976665
-0.0406508	-0.0735226	-0.3282984	-0.1938236	-0.250804	0.22205818	0.81023099
-0.0302085	-0.0799165	0.28326051	0.27662187	0.08403133	-0.0555311	-0.1626149
-0.058529	-0.1841377	0.04025506	0.30438765	-0.236963	0.13075637	0.09977365
-0.0117554	-0.0524305	0.12412495	0.07352819	0.13959856	-0.2542779	-0.168547
-0.0369056	0.08696495	0.09263961	0.15151857	0.20077548	0.0498518	-0.0380812
0.00023304	0.11289699	-0.1266433	-0.0131389	0.06239639	0.14959259	0.55177179
0.00045586	0.06137453	0.16037993	0.16335788	0.00399561	0.01518635	-0.2046714
0.02062496	-0.0450073	-0.0893609	-0.1090408	0.04829432	0.07295675	0.01940778
0.16784915	-0.0532691	-0.220428	-0.0821399	-0.1798038	0.06047862	0.58358604
1	0.00019757	-0.0250577	-0.0043467	-0.0469405	-0.0142	-0.0096616
0.00019757	1	-0.0024535	0.05770118	0.20587025	0.08534792	-0.0397704
-0.0250577	-0.0024535	1	0.2672722	0.09471997	-0.1454408	-0.3233042
-0.0043467	0.05770118	0.2672722	1	0.09773035	0.05131898	-0.158
-0.0469405	0.20587025	0.09471997	0.09773035	1	-0.1545328	-0.1831888
-0.0142	0.08534792	-0.1454408	0.05131898	-0.1545328	1	0.18699024
-0.0096616	-0.0397704	-0.3233042	-0.158	-0.1831888	0.18699024	1
-0.078763	0.03383292	-0.2952075	-0.2535905	0.0632745	-0.0859153	0.15934949
-0.0114703	-0.1255172	-0.3324658	-0.1463097	-0.190232	0.24509102	0.66449228
0.06215254	0.02465214	-0.0497039	-0.0053908	-0.0437683	0.07601869	0.40304718
0.04175902	0.18682381	0.20844787	0.32146109	0.08335427	0.18803409	-0.1309436
0.00634429	-0.1070396	-0.0120366	0.03653858	-0.0554514	-0.1670699	-0.0542567
0.04861185	0.01681661	-0.022787	-0.0123676	-0.0080923	0.0532029	0.38323767
0.00439849	-0.0062884	-0.118556	-0.0394489	-0.0610417	0.31476991	0.0360608
0.04728706	0.00319859	-0.0224597	-0.0142023	-0.0074159	0.06759778	0.37325621
-0.0569037	0.00856304	-0.4207249	-0.2467287	-0.1446254	0.06056762	0.48475099
0.022325	-0.0052039	0.11584938	0.14105746	0.01617016	-0.0095344	-0.1118676
0.01123982	0.08273583	-0.358463	-0.3390428	-0.2345646	0.30411699	0.25745692
-0.0731745	-0.2429669	-0.2099755	-0.0754525	-0.2001674	0.06378386	0.46978271
-0.0588936	-0.0182869	0.23939449	0.4210588	0.06560311	-0.0222816	-0.232735
-0.0305515	-0.0601243	-0.2104833	-0.2904786	-0.0873893	-0.0889261	0.19689188
0.02325238	-4.06E-05	-0.0694075	-0.083869	-0.0253122	0.07136621	-0.0280171
-0.0672056	-0.0592648	-0.1716049	-0.3941508	-0.0335097	-0.0297333	0.18252468
-0.0302792	-0.1755781	-0.1672667	-0.0486805	-0.2155713	0.00153561	0.54987831
-0.0290751	0.38221043	0.08549498	0.15255874	0.09195236	0.2134437	-0.066261
-0.0171898	-0.0337969	-0.036426	-0.0020537	0.000605	0.00261	0.0018842
-0.0338391	0.00796176	-0.3696074	-0.2501288	-0.0959778	0.18928517	0.67247091
0.00740594	-0.0134249	-0.1571515	-0.2006914	-0.1047834	0.09947823	0.25406348
-0.0148037	-0.0132533	0.03409116	0.05505884	0.00326523	-0.0036607	-0.0268314
-0.0385671	-0.0368549	-0.3559579	-0.195711	-0.2016014	0.23443048	0.84790783
-0.0294592	-0.0203171	-0.2937403	-0.460582	0.01576347	0.05733786	0.19831958

-0.0464736	-0.2553432	0.19853436	0.07908083	-0.0356338	-0.2813209	-0.1808562
0.11557261	-0.0870607	0.0716317	0.15230791	-0.0220694	-0.101452	-0.1224938
-0.019817	0.04394392	-0.152008	-0.0065	-0.188854	0.30904835	0.17952394
0.04914166	0.01484196	-0.0208844	-0.0135496	-0.0066858	0.05087285	0.38030959
0.07540538	-0.0164598	0.1005032	0.04209589	-0.0052309	-0.1766026	-0.1994793
0.00798342	-0.1115497	0.20908139	0.00547869	0.07727887	-0.220375	-0.1766836
0.04730657	0.01816532	-0.0265638	-0.0102207	-0.0073979	0.05880653	0.39057686
0.05935533	-0.1816486	-0.0866998	-0.0145377	-0.03142	-0.0699712	0.03884061
0.040691	-0.0025325	-0.0820008	-0.0687487	-0.0008109	0.00510588	-0.0365112
-0.0186805	-0.0097384	-0.3687049	-0.3981857	0.06482465	0.08840804	0.33811869
-0.1102459	-0.1688503	-0.1628691	-0.0564709	-0.0692491	0.06196618	0.43047338
-0.0347652	-0.1187252	-0.289989	-0.1549849	-0.223615	0.07613421	0.71844973
-0.0497552	-0.0054839	0.10511076	0.16280249	-0.0030156	-0.1426042	0.08172533
0.03108603	0.0440125	-0.2957507	-0.1970979	-0.1575179	0.17346879	0.2635378
0.04815726	0.01521748	-0.0254349	-0.0127584	-0.0092887	0.05541793	0.39085103
-0.0839023	-0.121471	-0.3117593	-0.3027089	-0.0172292	0.16216764	0.43057947
-0.0408163	0.00592243	0.13682302	0.17146549	-0.1255928	-0.0272912	-0.1837687
0.0211392	0.07198949	-0.2082758	-0.0933736	-0.0352101	0.08490576	0.2284763
-0.0572382	-0.1078486	-0.3819734	-0.2623973	-0.1351458	0.14099462	0.63346184
0.01079019	-0.2954645	0.07764847	0.07704141	-0.085304	-0.1076926	-0.1041191
-0.0208671	-0.1640245	-0.2153518	-0.0924024	-0.2618909	0.18775573	0.3510399
-0.0201937	0.03214053	0.19305169	0.15353886	-0.0330084	-0.0393698	-0.0437291
-0.0298364	-0.1639779	-0.1457882	-0.0712555	-0.1955073	0.02847672	0.5962217
-0.0467312	-0.0567313	-0.1601486	-0.2024646	-0.1424814	-0.0865682	0.04361083
-0.0825127	-0.0712062	0.09350692	0.08590147	0.08055969	-0.1917837	-0.158682
-0.0233753	-0.0193893	-0.2491293	-0.2427029	-0.1870279	0.18789187	0.20953387
-0.0390233	-0.1510303	0.06139075	0.07116214	-0.0885006	-0.0712047	-0.0271895
-0.1058525	-0.0372042	-0.2310545	-0.1532974	-0.0819969	0.15950951	0.33572287

PDPK1	GK	SLC27A5	FABP6	RXRB	APOA5	PLIN4
-0.3961516	-0.2881527	-0.0561427	0.34527758	-0.0444228	-0.0428588	-0.0843185
-0.0964069	-0.0465161	-0.0120759	0.01443056	-0.0362883	-0.0039477	-0.0045751
0.24170278	0.767012	0.13759091	-0.1628133	-0.0200656	0.11322572	0.03346701
0.08831051	0.0694413	0.01440373	-0.2422451	-0.0315142	-0.0135774	0.15508783
0.29469209	0.73107896	0.16134863	-0.150421	0.03598232	0.14082019	0.08976907
0.0128224	0.0695314	-0.0755516	0.11854659	-0.0161433	-0.0408087	-0.0310618
-0.0586218	0.23279365	0.06479976	-0.0819367	0.05802648	0.0771467	-0.0140053
0.136992	-0.152004	-0.0622331	-0.0543223	-0.03862	-0.0258044	-0.0087114
-0.0010169	0.09682226	-0.0725998	0.05374414	-0.1206216	-0.0377497	-0.0364109
0.1300219	0.29961575	0.52848026	0.16374266	-0.1718209	0.52373896	0.06036387
-0.3575428	-0.2639404	-0.0450806	0.09996635	-0.0471344	-0.0272587	-0.0620882
-0.0100158	-0.0069981	-0.0014273	-0.0587584	-0.0888356	-0.0022158	0.11551192
0.17223613	0.5864994	0.03128378	-0.0626701	0.04157649	-0.0205669	0.05252742
-0.078763	-0.0114703	0.06215254	0.04175902	0.00634429	0.04861185	0.00439849
0.03383292	-0.1255172	0.02465214	0.18682381	-0.1070396	0.01681661	-0.0062884
-0.2952075	-0.3324658	-0.0497039	0.20844787	-0.0120366	-0.022787	-0.118556
-0.2535905	-0.1463097	-0.0053908	0.32146109	0.03653858	-0.0123676	-0.0394489
0.0632745	-0.190232	-0.0437683	0.08335427	-0.0554514	-0.0080923	-0.0610417
-0.0859153	0.24509102	0.07601869	0.18803409	-0.1670699	0.0532029	0.31476991
0.15934949	0.66449228	0.40304718	-0.1309436	-0.0542567	0.38323767	0.0360608
1	0.35189862	-0.0697533	-0.2673727	0.10343228	-0.0507951	0.04211035
0.35189862	1	-0.0028215	-0.158173	-0.0451591	-0.0196234	0.08817505
-0.0697533	-0.0028215	1	-0.0086522	-0.0615621	0.98641924	0.02644762
-0.2673727	-0.158173	-0.0086522	1	-0.2415525	-0.0268269	-0.0462104
0.10343228	-0.0451591	-0.0615621	-0.2415525	1	-0.0678787	0.01879779
-0.0507951	-0.0196234	0.98641924	-0.0268269	-0.0678787	1	0.01540626
0.04211035	0.08817505	0.02644762	-0.0462104	0.01879779	0.01540626	1
-0.0530356	0.00459354	0.95924035	-0.0320631	-0.0503631	0.96051244	0.02026276
0.66326285	0.62049733	0.04610786	-0.282832	0.1058187	0.03823888	0.0950314
-0.159844	-0.1050123	-0.0431014	0.07628355	0.0265939	-0.0221185	-0.0587109
0.1326506	0.23755639	0.12576819	-0.1691579	-0.0627965	0.06131702	0.1747278
0.32285447	0.65328847	-0.0176858	-0.216954	0.0567503	-0.0143523	-0.0204359
-0.0584612	-0.2171107	-0.0559214	0.18867954	-0.1901071	-0.0200478	-0.0083439
0.53451276	0.35659852	0.01785008	-0.324743	0.18998727	0.01278294	0.04311345
0.07249355	-0.0427297	0.01151122	-0.041023	-0.0493097	0.00287899	0.05422147
0.20309141	0.08774444	-0.0147139	-0.244843	0.17753277	-0.0276963	-0.0318252
0.17238981	0.59543165	0.01401849	-0.1606455	0.0874521	0.00677787	-0.0220407
-0.1045087	-0.1467426	-0.0225812	0.28210273	-0.2158848	-0.0206594	0.13604773
-0.0066714	-0.0167904	-0.0044904	-0.0414263	0.07738196	-0.0056274	0.72382834
0.48835636	0.72126803	0.17541931	-0.2265779	-0.05791	0.16587548	0.07658004
0.21963924	0.28894502	0.01089208	-0.0974155	-0.0141459	-0.0080754	0.02174535
-0.0236173	-0.0307008	-0.0086328	-0.0113334	0.13061422	-0.0111625	0.69426505
0.23785251	0.71621613	0.07628961	-0.1406928	0.05507075	0.04545569	0.06361939
0.48840924	0.30531437	-0.0152936	-0.3188759	-0.1238212	-0.0248921	0.04858085

-0.0489194	-0.1241945	-0.0741556	-0.1586668	0.13373229	-0.0343513	-0.0099858
-0.0623126	-0.0164184	0.01135652	0.00641277	0.38660604	-0.0112193	0.04507786
0.02858293	0.16442241	0.14975863	0.19824273	-0.1462335	0.12064331	0.28498566
-0.0517694	-0.017968	0.98665144	-0.0280614	-0.0616496	0.99866232	0.01467102
-0.2859204	-0.3291387	-0.0201331	0.0412429	0.19289109	-0.0343144	0.00986983
-0.1873896	-0.2649914	-0.0817524	-0.0986329	0.31176169	-0.0488941	-0.0492755
-0.050888	-0.0140707	0.98515037	-0.0265443	-0.0699747	0.99904203	0.01839934
0.01694897	0.07556948	0.03406673	-0.168298	0.14994362	0.00313095	0.32030322
0.02449391	-0.0440747	0.02631759	-0.0780723	0.05231924	0.00256411	0.45629187
0.27987944	0.31883356	-0.0123032	-0.2382013	-0.1059037	-0.0202137	-0.0021906
0.44999543	0.63092969	-0.0269802	-0.1535299	-0.0097609	-0.0069905	-0.0042647
0.24720524	0.68268352	0.0337022	-0.1873026	0.00846046	0.01395583	0.00748653
0.09600098	-0.0177631	0.05391468	0.00855645	0.07920528	0.06129128	0.06481919
0.19612437	0.2506811	0.19473265	-0.0541346	0.02384128	0.14028938	0.31735647
-0.0512056	-0.0132095	0.98656091	-0.0268318	-0.0679098	0.99910596	0.01544716
0.44260964	0.48734995	0.19153227	-0.208287	-0.1791415	0.20594628	0.04594787
-0.0767298	-0.2023821	0.06375105	-0.0322026	0.19347327	0.02972371	0.13657536
0.16933558	0.27744199	0.00518459	-0.0287128	-0.0680952	-0.0077995	0.08047511
0.5177654	0.71778759	-0.0089324	-0.2374463	-0.0008894	-0.0217507	0.01599964
0.22153392	0.24702118	-0.0476292	-0.090013	0.19784703	-0.0369347	-0.0347974
0.19992519	0.4784595	0.03536389	-0.1527615	0.20236609	0.00551434	0.22703706
-0.1306676	-0.1534821	0.22746567	0.09483721	-0.1231777	0.24082728	-0.0085154
0.1226438	0.5532881	0.03209363	-0.1441352	0.07860151	0.0219847	0.01458283
0.18942755	0.05477481	-0.0330385	-0.2359198	0.12970406	-0.0345059	0.22227859
0.20479871	-0.0698671	-0.0844123	-0.2740769	0.34880556	-0.050118	-0.0546927
0.45231593	0.35901683	0.08966851	-0.2323014	0.00047879	0.07818787	0.16883438
-0.0590075	-0.0226987	-0.0133703	-0.109239	0.10773424	-0.0104955	0.28354831
0.48247585	0.4805087	-0.0315809	-0.1810493	0.06889978	-0.025591	0.11211748

APOA2	PPARA	FABP7	SLC27A4	SLC27A2	SCD	CPT1A
-0.0426137	-0.3916332	0.17461914	-0.3749133	-0.1207742	0.22003117	-0.2358797
-0.0052138	-0.0849679	-0.0106361	-0.0259574	-0.0600194	0.00911221	-0.0832411
0.11168736	0.57326176	-0.1343003	0.25915954	0.65920175	-0.2194823	0.22665495
-0.0007511	0.16937544	-0.0508516	0.18595965	0.07559305	-0.1536681	0.3250108
0.14225503	0.59576735	-0.1166141	0.24074672	0.62884401	-0.2495347	0.25053133
-0.0089136	-0.0276388	0.04832058	-0.3194843	0.16221768	0.28359116	0.0713875
0.09658088	0.05174028	0.05618786	-0.0997663	0.45190615	0.04220748	0.03740769
-0.037438	-0.0105695	0.09778553	-0.2937454	-0.0885928	0.28905901	0.0305378
-0.0445905	-0.0466203	0.00941359	-0.2332084	-0.0338692	0.0845879	-0.1437357
0.52073377	0.26084227	-0.0461225	0.08698709	0.25136283	-0.0498514	-0.0145987
-0.0158394	-0.39737	0.09561967	-0.0903474	-0.2541938	0.15614811	-0.310168
-0.0004628	-0.0246174	-0.042039	0.15564377	-0.0661098	-0.0673619	0.02137435
-0.0108518	0.46798953	-0.0703605	0.17891976	0.40667086	-0.1278558	0.14255246
0.04728706	-0.0569037	0.022325	0.01123982	-0.0731745	-0.0588936	-0.0305515
0.00319859	0.00856304	-0.0052039	0.08273583	-0.2429669	-0.0182869	-0.0601243
-0.0224597	-0.4207249	0.11584938	-0.358463	-0.2099755	0.23939449	-0.2104833
-0.0142023	-0.2467287	0.14105746	-0.3390428	-0.0754525	0.4210588	-0.2904786
-0.0074159	-0.1446254	0.01617016	-0.2345646	-0.2001674	0.06560311	-0.0873893
0.06759778	0.06056762	-0.0095344	0.30411699	0.06378386	-0.0222816	-0.0889261
0.37325621	0.48475099	-0.1118676	0.25745692	0.46978271	-0.232735	0.19689188
-0.0530356	0.66326285	-0.159844	0.1326506	0.32285447	-0.0584612	0.53451276
0.00459354	0.62049733	-0.1050123	0.23755639	0.65328847	-0.2171107	0.35659852
0.95924035	0.04610786	-0.0431014	0.12576819	-0.0176858	-0.0559214	0.01785008
-0.0320631	-0.282832	0.07628355	-0.1691579	-0.216954	0.18867954	-0.324743
-0.0503631	0.1058187	0.0265939	-0.0627965	0.0567503	-0.1901071	0.18998727
0.96051244	0.03823888	-0.0221185	0.06131702	-0.0143523	-0.0200478	0.01278294
0.02026276	0.0950314	-0.0587109	0.1747278	-0.0204359	-0.0083439	0.04311345
1	0.0368693	-0.0310035	0.0611777	-0.0061447	-0.0289102	0.02004353
0.0368693	1	-0.1737538	0.26548484	0.51201397	-0.1838227	0.62283171
-0.0310035	-0.1737538	1	-0.2058197	-0.0857059	0.11632969	-0.1430895
0.0611777	0.26548484	-0.2058197	1	0.05425318	-0.3623418	0.36486762
-0.0061447	0.51201397	-0.0857059	0.05425318	1	-0.1320312	0.28659764
-0.0289102	-0.1838227	0.11632969	-0.3623418	-0.1320312	1	-0.2337433
0.02004353	0.62283171	-0.1430895	0.36486762	0.28659764	-0.2337433	1
0.00211836	0.099696	-0.0273808	0.05249321	-0.0822185	0.00475733	0.09648458
-0.0320114	0.21762598	-0.1127607	0.16553642	0.0670449	-0.3741189	0.14637511
0.02290202	0.43183722	-0.0853304	0.08432717	0.65754815	-0.1805204	0.34306493
-0.0279738	-0.2132689	-0.0067429	-0.031876	-0.2634707	0.16748471	-0.3248837
-0.0075775	-0.003056	-0.0128814	0.0318938	-0.0209612	0.03716563	0.00619604
0.16383426	0.73265337	-0.156466	0.31906696	0.62574359	-0.2170518	0.49906041
0.00845393	0.30874274	-0.0639907	0.24555024	0.24721659	-0.1531285	0.21417477
-0.0119421	-0.0212231	-0.0044885	-0.0296897	-0.0373353	0.10586269	-0.0158219
0.05235568	0.54439581	-0.1185497	0.31558194	0.54202699	-0.3149412	0.26881051
-0.0274009	0.4622176	-0.175219	0.49580964	0.11315479	-0.1461098	0.52106842

-0.0417483	-0.1006801	0.11686671	-0.3148238	0.18890431	0.10967385	0.07616843
-0.0112691	-0.0262564	0.0138693	0.01390877	-0.105458	-0.0691895	0.08752712
0.13366538	0.16152127	-0.1489943	0.21277037	0.05362021	-0.0362563	0.13795201
0.97206277	0.03736971	-0.0221834	0.05830428	-0.0153518	-0.0233061	0.01449346
-0.0347744	-0.301303	0.02409561	-0.0506403	-0.2211701	0.09748564	-0.231766
-0.0552089	-0.2194195	0.01773388	-0.2378303	-0.0867545	-0.040793	-0.1687423
0.95876266	0.04377385	-0.0215565	0.06231159	-0.0116502	-0.0201346	0.01129402
0.08275227	0.08055902	-0.0500522	0.12986009	0.11634514	-0.0913875	0.16477525
0.00394821	0.04868627	-0.0447642	0.10726174	-0.0938207	-0.0108739	0.03992784
-0.0204503	0.26856185	-0.1714888	0.35118832	0.1503016	-0.2107724	0.17120575
0.00035431	0.55961581	-0.081714	-0.0437913	0.7268075	-0.0135828	0.34454291
0.01366705	0.53954883	-0.1261	0.21450197	0.65133929	-0.2174037	0.28962123
0.04674271	0.04995552	-0.015004	-0.0510975	0.093516	0.08902117	-0.0345193
0.13521315	0.3531784	-0.1461062	0.52534674	0.00962408	-0.1892337	0.31145879
0.96765219	0.03995982	-0.0233392	0.062287	-0.0115663	-0.0216687	0.01275631
0.22162568	0.46069829	-0.0905132	0.13171889	0.45474735	-0.0919671	0.34027946
0.03956784	-0.1042891	-0.012883	0.02219314	-0.083448	-0.0214508	-0.0180998
9.03E-07	0.306813	-0.0631199	0.06910201	0.27763672	-0.0908414	0.05860721
-0.0018972	0.67706036	-0.1431859	0.22997587	0.61623085	-0.2047406	0.485364
-0.004639	0.22204614	0.03538034	-0.1714476	0.32518065	0.02531975	0.35218742
0.00370952	0.44576262	-0.1356263	0.26067661	0.41325381	-0.2009649	0.38412159
0.23772405	-0.1374748	0.11182476	-0.0906871	-0.1272499	0.27999836	-0.1177437
0.04018466	0.37672357	-0.0709048	0.08977055	0.54390131	-0.177295	0.31005542
-0.0338204	0.28734322	-0.0942233	0.16081232	0.07356572	-0.1195785	0.26896304
-0.0354243	0.05991324	0.0564761	-0.2043861	0.18509192	0.03796403	0.17146797
0.09415509	0.57048128	-0.1807074	0.41388019	0.22928551	-0.0977896	0.65885275
-0.0140474	-0.0026014	0.06651062	-0.0250409	0.14174933	-0.0041664	0.10024328
-0.0055582	0.56017079	-0.1117458	0.20527716	0.56627954	-0.1336247	0.52924282

ACSBG1	SCD5	CYP4A11	PLTP	ADIPOQ	ACOX1	ACSL6
-0.0668969	-0.268243	-0.0513418	0.09527797	-0.0591563	-0.3617371	-0.2341075
-0.0104311	-0.0314069	-0.0389765	0.02242799	-0.0071935	-0.076246	-0.0359063
-0.0241919	0.18057151	0.64246509	-0.098961	-0.0173237	0.73917787	0.30982154
0.08818939	-0.0733852	0.07066137	-0.2578443	0.01531389	0.05958804	0.03205997
-0.0420459	0.22043423	0.61015763	-0.0845911	0.00564303	0.70378269	0.30853388
-0.086278	-0.2740956	0.22861613	-0.1302079	-0.0391242	0.0153479	-0.1248066
-0.0311878	-0.1185252	0.33926966	-0.1443878	-0.0237674	0.14615864	-0.017595
0.01157215	-0.0703266	-0.1512548	-0.0205932	0.0666859	-0.0905919	-0.1349214
-0.0589473	-0.0355825	-0.0445173	0.07805209	-0.0170975	-0.0231514	-0.098053
-0.0278481	0.06292317	0.24408015	0.20736782	0.00085993	0.4016315	0.09496482
0.06602863	-0.2355939	-0.1861062	0.1907993	-0.0124747	-0.3287432	-0.1888221
0.03241509	-0.0024364	-0.0442577	0.03815046	0.05192187	-0.012371	0.07155706
0.04826549	0.14217043	0.45652874	-0.0255541	0.01455881	0.51644211	0.29045678
0.02325238	-0.0672056	-0.0302792	-0.0290751	-0.0171898	-0.0338391	0.00740594
-4.06E-05	-0.0592648	-0.1755781	0.38221043	-0.0337969	0.00796176	-0.0134249
-0.0694075	-0.1716049	-0.1672667	0.08549498	-0.036426	-0.3696074	-0.1571515
-0.083869	-0.3941508	-0.0486805	0.15255874	-0.0020537	-0.2501288	-0.2006914
-0.0253122	-0.0335097	-0.2155713	0.09195236	0.000605	-0.0959778	-0.1047834
0.07136621	-0.0297333	0.00153561	0.2134437	0.00261	0.18928517	0.09947823
-0.0280171	0.18252468	0.54987831	-0.066261	0.0018842	0.67247091	0.25406348
0.07249355	0.20309141	0.17238981	-0.1045087	-0.0066714	0.48835636	0.21963924
-0.0427297	0.08774444	0.59543165	-0.1467426	-0.0167904	0.72126803	0.28894502
0.01151122	-0.0147139	0.01401849	-0.0225812	-0.0044904	0.17541931	0.01089208
-0.041023	-0.244843	-0.1606455	0.28210273	-0.0414263	-0.2265779	-0.0974155
-0.0493097	0.17753277	0.0874521	-0.2158848	0.07738196	-0.05791	-0.0141459
0.00287899	-0.0276963	0.00677787	-0.0206594	-0.0056274	0.16587548	-0.0080754
0.05422147	-0.0318252	-0.0220407	0.13604773	0.72382834	0.07658004	0.02174535
0.00211836	-0.0320114	0.02290202	-0.0279738	-0.0075775	0.16383426	0.00845393
0.099696	0.21762598	0.43183722	-0.2132689	-0.003056	0.73265337	0.30874274
-0.0273808	-0.1127607	-0.0853304	-0.0067429	-0.0128814	-0.156466	-0.0639907
0.05249321	0.16553642	0.08432717	-0.031876	0.0318938	0.31906696	0.24555024
-0.0822185	0.0670449	0.65754815	-0.2634707	-0.0209612	0.62574359	0.24721659
0.00475733	-0.3741189	-0.1805204	0.16748471	0.03716563	-0.2170518	-0.1531285
0.09648458	0.14637511	0.34306493	-0.3248837	0.00619604	0.49906041	0.21417477
1	-0.0743645	-0.0703176	0.07417594	-0.01151	-0.0051694	0.02861407
-0.0743645	1	0.05334521	-0.1607001	0.04218096	0.24074979	0.16137307
-0.0703176	0.05334521	1	-0.1893112	-0.0241449	0.55677464	0.24816004
0.07417594	-0.1607001	-0.1893112	1	0.02161154	-0.156497	-0.0944249
-0.01151	0.04218096	-0.0241449	0.02161154	1	-0.0074384	0.01409756
-0.0051694	0.24074979	0.55677464	-0.156497	-0.0074384	1	0.40888692
0.02861407	0.16137307	0.24816004	-0.0944249	0.01409756	0.40888692	1
-0.0101624	-0.0074153	-0.009446	0.04521396	0.91854287	-0.039483	-0.009257
-0.0174847	0.25472188	0.62083111	-0.0931751	0.00679591	0.67916399	0.30566971
0.13994946	0.23723918	0.0686154	-0.1391026	0.0203065	0.45665442	0.29944335

-0.0506718	-0.1309272	0.19575183	-0.2290803	0.08396363	-0.1034447	-0.0131308
0.07315642	-0.2227749	0.02535744	-0.1134498	-0.0113882	-0.2121793	-0.0724466
0.0786666	-0.0618705	0.11169314	0.02809448	0.010894	0.15758383	0.05830083
0.00156575	-0.0266311	0.0080933	-0.0236022	-0.0050102	0.16392468	-0.0070672
0.08101598	-0.0516346	-0.172647	0.09371376	0.08634594	-0.3094385	-0.1061862
-0.0582699	0.20930141	-0.079911	-0.0705326	0.01767438	-0.2703147	-0.1774442
0.00267633	-0.0273876	0.01063568	-0.0145109	-0.0058021	0.17111294	-0.0065805
0.0428604	0.04547721	0.13912721	-0.1365328	0.38089695	0.0612358	0.14688613
0.21926174	0.01234174	-0.0853612	0.01103302	0.51520769	-0.0035141	0.02706003
-0.0409244	0.33432624	0.10268496	-0.0297296	0.04344654	0.41510832	0.24715498
-0.0839862	0.06593547	0.66833961	-0.2055575	-0.0553715	0.66444005	0.24734933
-0.0348827	0.16455146	0.64953932	-0.1321954	0.0068862	0.67637067	0.29577352
0.0013536	0.01822489	0.05226834	0.03064386	0.05623086	0.02975507	0.06650067
0.10048161	0.04722331	0.03559337	0.0324566	0.10690836	0.2377076	0.1264316
0.00173303	-0.0273297	0.01066711	-0.0203106	-0.0060946	0.16924157	-0.005855
0.00121924	0.20405815	0.36266306	-0.1631468	-0.0223296	0.6217799	0.26345905
0.02045014	0.03500301	-0.0973774	-0.1025802	0.01227746	-0.2986622	-0.0703016
8.10E-05	0.07467562	0.21380164	0.00923661	0.04181926	0.34523448	0.14989182
-0.0185721	0.29761091	0.5505479	-0.2343954	-0.0023447	0.76458334	0.34691294
0.01849474	-0.1621596	0.31752455	-0.3373696	-0.0524908	0.11264434	0.02950895
0.04537911	0.02412608	0.49973133	-0.2094058	0.13257221	0.40554421	0.1916426
-0.0151302	-0.1694496	-0.0431632	0.06961925	-0.013479	-0.1063294	-0.0810452
-0.0586156	0.06658627	0.88719294	-0.1664092	-0.00873	0.51979635	0.2203186
-0.0557412	0.20380141	0.06002319	-0.1818529	0.32587012	0.19175105	0.18134411
-0.0649715	0.0391573	0.07216848	-0.19634	0.00555869	0.00844351	-0.0572345
0.13194349	0.02052856	0.26461707	-0.1866841	-0.0181162	0.4367247	0.22214723
-0.0299871	-0.0397415	0.22348275	-0.113996	0.38854469	0.01133931	0.06397187
0.00473652	0.1531301	0.54355685	-0.2156747	-0.0227311	0.61099157	0.28798888

PLIN1	ACAA1	ACSL3	CD36	CPT1B	FABP3	CYP7A1
0.00186826	-0.2718777	-0.4752871	0.2119116	0.1020781	0.01852679	-0.041902
-0.0106968	-0.0508101	-0.059093	-0.0047645	-0.0203718	-0.0510027	-0.0028154
-0.0351737	0.84870732	0.188907	-0.1549219	-0.1309509	0.16273771	0.1106209
0.00394715	-0.0333389	0.25410135	0.29905613	0.17006525	0.06178096	-0.0121746
-0.0060732	0.80130924	0.18130362	-0.152621	-0.1275456	0.21334355	0.13918922
0.01790474	-0.1369281	-0.2227233	0.26480918	-0.0084242	0.09786583	-0.0361957
-0.0062888	0.12349254	-0.2165928	0.18720512	0.01511576	0.00950942	0.07923807
0.07591686	-0.2215697	0.00171484	0.15167801	-0.0472341	-0.1545023	-0.0250404
0.00188443	-0.0430086	-0.1370874	-0.1159945	0.03156742	0.03403505	-0.0399192
-0.0105086	0.38542909	-0.0166559	-0.2289291	-0.1265229	0.20470272	0.52478
-0.0062211	-0.281646	-0.1724961	0.14342416	0.06521577	-0.136454	-0.0244848
0.04368936	0.01525333	0.20643933	-0.0813281	-0.0090893	0.06086393	-0.0022638
0.05298595	0.62027032	0.15242244	-0.1570208	0.06170643	0.04307571	-0.0231245
-0.0148037	-0.0385671	-0.0294592	-0.0464736	0.11557261	-0.019817	0.04914166
-0.0132533	-0.0368549	-0.0203171	-0.2553432	-0.0870607	0.04394392	0.01484196
0.03409116	-0.3559579	-0.2937403	0.19853436	0.0716317	-0.152008	-0.0208844
0.05505884	-0.195711	-0.460582	0.07908083	0.15230791	-0.0065	-0.0135496
0.00326523	-0.2016014	0.01576347	-0.0356338	-0.0220694	-0.188854	-0.0066858
-0.0036607	0.23443048	0.05733786	-0.2813209	-0.101452	0.30904835	0.05087285
-0.0268314	0.84790783	0.19831958	-0.1808562	-0.1224938	0.17952394	0.38030959
-0.0236173	0.23785251	0.48840924	-0.0489194	-0.0623126	0.02858293	-0.0517694
-0.0307008	0.71621613	0.30531437	-0.1241945	-0.0164184	0.16442241	-0.017968
-0.0086328	0.07628961	-0.0152936	-0.0741556	0.01135652	0.14975863	0.98665144
-0.0113334	-0.1406928	-0.3188759	-0.1586668	0.00641277	0.19824273	-0.0280614
0.13061422	0.05507075	-0.1238212	0.13373229	0.38660604	-0.1462335	-0.0616496
-0.0111625	0.04545569	-0.0248921	-0.0343513	-0.0112193	0.12064331	0.99866232
0.69426505	0.06361939	0.04858085	-0.0099858	0.04507786	0.28498566	0.01467102
-0.0119421	0.05235568	-0.0274009	-0.0417483	-0.0112691	0.13366538	0.97206277
-0.0212231	0.54439581	0.4622176	-0.1006801	-0.0262564	0.16152127	0.03736971
-0.0044885	-0.1185497	-0.175219	0.11686671	0.0138693	-0.1489943	-0.0221834
-0.0296897	0.31558194	0.49580964	-0.3148238	0.01390877	0.21277037	0.05830428
-0.0373353	0.54202699	0.11315479	0.18890431	-0.105458	0.05362021	-0.0153518
0.10586269	-0.3149412	-0.1461098	0.10967385	-0.0691895	-0.0362563	-0.0233061
-0.0158219	0.26881051	0.52106842	0.07616843	0.08752712	0.13795201	0.01449346
-0.0101624	-0.0174847	0.13994946	-0.0506718	0.07315642	0.0786666	0.00156575
-0.0074153	0.25472188	0.23723918	-0.1309272	-0.2227749	-0.0618705	-0.0266311
-0.009446	0.62083111	0.0686154	0.19575183	0.02535744	0.11169314	0.0080933
0.04521396	-0.0931751	-0.1391026	-0.2290803	-0.1134498	0.02809448	-0.0236022
0.91854287	0.00679591	0.0203065	0.08396363	-0.0113882	0.010894	-0.0050102
-0.039483	0.67916399	0.45665442	-0.1034447	-0.2121793	0.15758383	0.16392468
-0.009257	0.30566971	0.29944335	-0.0131308	-0.0724466	0.05830083	-0.0070672
1	-0.0152373	-0.0243057	0.11266523	0.053736	-0.0033692	-0.0104496
-0.0152373	1	0.23224966	-0.2145887	-0.0882898	0.21190421	0.04462571
-0.0243057	0.23224966	1	-0.1044374	-0.1531221	-0.0411321	-0.0260233

0.11266523	-0.2145887	-0.1044374	1	0.04481511	-0.1300682	-0.0327975
0.053736	-0.0882898	-0.1531221	0.04481511	1	-0.0070182	-0.0097594
-0.0033692	0.21190421	-0.0411321	-0.1300682	-0.0070182	1	0.12085369
-0.0104496	0.04462571	-0.0260233	-0.0327975	-0.0097594	0.12085369	1
0.11855487	-0.2525605	-0.1205636	0.14533459	0.05990245	-0.2499276	-0.0352932
0.04336512	-0.1890061	-0.2751512	0.2150209	0.06544418	-0.1742496	-0.0486881
-0.0113175	0.05370985	-0.0254675	-0.0382682	-0.013715	0.12048271	0.99747713
0.37678828	0.05125159	0.06678956	0.19606574	0.09883418	0.07703663	0.01614388
0.53451796	-0.0356368	0.11219802	0.0354651	0.04554399	-0.0470965	0.00118134
-0.0178068	0.34254982	0.58609609	-0.2173488	-0.2621226	-0.0405539	-0.0207368
-0.0313642	0.49929134	0.16269322	0.16907765	-0.1806865	0.13341365	-0.0079887
-0.0175212	0.76720645	0.23275131	-0.0078992	-0.1201968	0.10545623	0.01181363
0.05410482	0.01866784	-0.095219	0.06152954	-0.0892677	-0.0742803	0.05891754
0.0836212	0.27921219	0.35078232	-0.2499051	0.12900217	0.23439483	0.13847034
-0.0120443	0.0521992	-0.0243473	-0.0380459	-0.0132674	0.12326744	0.9988656
-0.0759369	0.42900532	0.50788166	0.04996833	-0.3107728	0.20361193	0.20893658
0.02746414	-0.1953563	-0.2564451	0.0575878	0.24770968	0.14880481	0.03000982
0.02949899	0.26443219	0.13555021	-0.0640397	-0.0379032	0.07789183	-0.0075441
-0.0428996	0.72039867	0.47853778	-0.0471519	-0.2225281	0.17156442	-0.0195862
0.01110942	-0.0217333	0.03352496	0.33440362	0.30464849	0.03668691	-0.0300267
0.13138964	0.46014167	0.14334965	0.07036832	0.21075167	0.22158179	0.00479075
0.00843577	-0.1715268	-0.1382123	0.06438313	-0.0293731	-0.0292567	0.24000663
0.00251974	0.66853704	0.07730379	0.18446304	0.03790264	0.11009904	0.023741
0.26877896	0.08572246	0.29765122	0.23425694	-0.1159556	0.01115419	-0.0317586
0.01111383	-0.1205343	-0.0383946	0.28826488	-0.1158113	-0.141113	-0.0457966
-0.039778	0.2856845	0.5031355	-0.0132591	0.00801071	0.32144621	0.07864117
0.37583318	-0.0322775	-0.0366102	0.65524616	0.08272059	0.02104774	-0.0099861
-0.0462051	0.49414186	0.26882365	0.08189415	-0.1906475	0.3052228	-0.0241544

CPT1C	PPARD	APOC3	RXRG	UCP1	ACSL4	EHHADH
0.09974077	0.28767735	-0.0442978	-0.0917775	-0.1031845	-0.3947396	-0.1050967
0.00929119	0.02419079	-0.0042835	-0.0362096	-0.0177238	-0.0049857	-0.0628078
-0.2164113	-0.1911449	0.12118232	0.05522595	-0.0468862	0.31699864	0.58221116
-0.1390696	-0.0511146	-0.0172142	0.15462351	0.16595761	-0.0777993	0.02081511
-0.2265228	-0.1775097	0.14740606	0.08710093	-0.0259706	0.32040565	0.57596335
-0.0898239	-0.0092928	-0.0390897	0.03305595	-0.0908845	-0.2752336	0.34747955
-0.1153263	-0.0007135	0.07874436	0.09943393	-0.0806215	-0.2253568	0.32055371
0.0730931	0.22521357	-0.0263998	0.08094604	0.01395011	-0.023419	-0.0375902
-0.1928381	0.03566595	-0.0387512	-0.1125661	-0.0613283	-0.010089	0.07718155
-0.1700182	-0.139966	0.5288043	-0.0340611	-0.0342974	0.18904254	0.25898407
0.40676393	0.16892974	-0.0294788	-0.1150479	-0.0106936	-0.1791798	-0.2970308
-0.0211661	-0.0799467	-0.0023431	0.02477657	0.1258256	0.25888894	-0.0626125
0.04956101	-0.1146425	-0.0163926	0.13720689	0.12566021	0.17964953	0.38468908
0.07540538	0.00798342	0.04730657	0.05935533	0.040691	-0.0186805	-0.1102459
-0.0164598	-0.1115497	0.01816532	-0.1816486	-0.0025325	-0.0097384	-0.1688503
0.1005032	0.20908139	-0.0265638	-0.0866998	-0.0820008	-0.3687049	-0.1628691
0.04209589	0.00547869	-0.0102207	-0.0145377	-0.0687487	-0.3981857	-0.0564709
-0.0052309	0.07727887	-0.0073979	-0.03142	-0.0008109	0.06482465	-0.0692491
-0.1766026	-0.220375	0.05880653	-0.0699712	0.00510588	0.08840804	0.06196618
-0.1994793	-0.1766836	0.39057686	0.03884061	-0.0365112	0.33811869	0.43047338
-0.2859204	-0.1873896	-0.050888	0.01694897	0.02449391	0.27987944	0.44999543
-0.3291387	-0.2649914	-0.0140707	0.07556948	-0.0440747	0.31883356	0.63092969
-0.0201331	-0.0817524	0.98515037	0.03406673	0.02631759	-0.0123032	-0.0269802
0.0412429	-0.0986329	-0.0265443	-0.168298	-0.0780723	-0.2382013	-0.1535299
0.19289109	0.31176169	-0.0699747	0.14994362	0.05231924	-0.1059037	-0.0097609
-0.0343144	-0.0488941	0.99904203	0.00313095	0.00256411	-0.0202137	-0.0069905
0.00986983	-0.0492755	0.01839934	0.32030322	0.45629187	-0.0021906	-0.0042647
-0.0347744	-0.0552089	0.95876266	0.08275227	0.00394821	-0.0204503	0.00035431
-0.301303	-0.2194195	0.04377385	0.08055902	0.04868627	0.26856185	0.55961581
0.02409561	0.01773388	-0.0215565	-0.0500522	-0.0447642	-0.1714888	-0.081714
-0.0506403	-0.2378303	0.06231159	0.12986009	0.10726174	0.35118832	-0.0437913
-0.2211701	-0.0867545	-0.0116502	0.11634514	-0.0938207	0.1503016	0.7268075
0.09748564	-0.040793	-0.0201346	-0.0913875	-0.0108739	-0.2107724	-0.0135828
-0.231766	-0.1687423	0.01129402	0.16477525	0.03992784	0.17120575	0.34454291
0.08101598	-0.0582699	0.00267633	0.0428604	0.21926174	-0.0409244	-0.0839862
-0.0516346	0.20930141	-0.0273876	0.04547721	0.01234174	0.33432624	0.06593547
-0.172647	-0.079911	0.01063568	0.13912721	-0.0853612	0.10268496	0.66833961
0.09371376	-0.0705326	-0.0145109	-0.1365328	0.01103302	-0.0297296	-0.2055575
0.08634594	0.01767438	-0.0058021	0.38089695	0.51520769	0.04344654	-0.0553715
-0.3094385	-0.2703147	0.17111294	0.0612358	-0.0035141	0.41510832	0.66444005
-0.1061862	-0.1774442	-0.0065805	0.14688613	0.02706003	0.24715498	0.24734933
0.11855487	0.04336512	-0.0113175	0.37678828	0.53451796	-0.0178068	-0.0313642
-0.2525605	-0.1890061	0.05370985	0.05125159	-0.0356368	0.34254982	0.49929134
-0.1205636	-0.2751512	-0.0254675	0.06678956	0.11219802	0.58609609	0.16269322

0.14533459	0.2150209	-0.0382682	0.19606574	0.0354651	-0.2173488	0.16907765
0.05990245	0.06544418	-0.013715	0.09883418	0.04554399	-0.2621226	-0.1806865
-0.2499276	-0.1742496	0.12048271	0.07703663	-0.0470965	-0.0405539	0.13341365
-0.0352932	-0.0486881	0.99747713	0.01614388	0.00118134	-0.0207368	-0.0079887
1	0.22985648	-0.0386625	0.13663491	0.19475952	-0.1953993	-0.3165194
0.22985648	1	-0.0523546	0.01407589	-0.0038344	-0.1976423	-0.1216685
-0.0386625	-0.0523546	1	0.00202193	0.00032526	-0.0184587	-0.003025
0.13663491	0.01407589	0.00202193	1	0.38050027	0.0215213	0.03724861
0.19475952	-0.0038344	0.00032526	0.38050027	1	-0.019698	-0.1069018
-0.1953993	-0.1976423	-0.0184587	0.0215213	-0.019698	1	0.13280301
-0.3165194	-0.1216685	-0.003025	0.03724861	-0.1069018	0.13280301	1
-0.1959956	-0.1399788	0.02051375	0.08164043	-0.059727	0.31565139	0.5766677
0.20763551	0.05560695	0.06465755	0.07602151	0.02123318	-0.1429075	0.12128952
-0.1436344	-0.1609987	0.14308202	0.09719062	0.14557578	0.150822	-0.0374052
-0.0381779	-0.0520115	0.99853471	0.01021319	0.00034537	-0.0171478	-0.0048018
-0.3575296	-0.2639963	0.21011085	0.05533697	-0.0438928	0.44046366	0.55915724
0.10958628	0.10476412	0.02459473	0.09590678	0.08725339	-0.29853	-0.1199627
-0.1450595	-0.1119885	-0.0029512	-0.0017917	0.09004534	0.21509598	0.19954918
-0.3344317	-0.2538107	-0.0165818	0.07555277	-0.0281616	0.41041466	0.67418523
-0.1797436	-0.0048485	-0.0368379	0.15482666	-0.0325967	-0.2310794	0.40572435
-0.1851555	-0.1541421	0.00872835	0.15310072	0.12335475	0.02408734	0.39963487
0.26438596	0.02736082	0.23780926	0.02947657	0.00232306	-0.1530882	-0.0728913
-0.1473814	-0.0648637	0.02599065	0.13477273	-0.0740577	0.12525377	0.55753561
0.04272229	-0.0764386	-0.036235	0.17338624	0.11766454	0.09886822	0.0491318
0.12527699	0.16674878	-0.05478	0.11831735	-0.0359966	-0.0925794	0.20142265
-0.3304056	-0.3001591	0.07811836	0.06065779	0.0719102	0.14740446	0.32407936
0.0574506	0.07717672	-0.0119976	0.31796569	0.15714991	-0.0755434	0.07541781
-0.3296255	-0.1882206	-0.022693	0.11578186	-0.0516201	0.12498809	0.69091397

PCK1	RXRA	PLIN5	APOA1	SCP2	SLC27A1	FABP2
-0.2289249	0.0481344	-0.2749759	-0.0432919	-0.3018309	0.10686011	-0.1556754
-0.0396967	-0.0599623	-0.0076209	-0.0044053	-0.0488665	-0.0367134	-0.0011264
0.80945237	0.10815239	0.20663042	0.11914094	0.43021477	-0.1651765	0.27384271
-0.0271354	-0.1579045	0.25983559	-0.0145835	0.08420648	0.11676555	0.02953004
0.73631881	0.12622491	0.23052332	0.1465074	0.42137216	-0.0786412	0.31829928
-0.0627752	0.11398718	-0.303839	-0.0385659	0.04276251	0.01068696	-0.0938445
0.16795848	0.02363279	-0.1491374	0.07915658	0.0641418	0.06380785	0.00830455
-0.1263646	0.08816373	-0.1044161	-0.0288915	0.00628156	0.01258192	0.02249648
-0.0715954	-0.2193242	-0.1484185	-0.037836	-0.0009544	-0.0519982	0.04405628
0.32216294	0.05053849	0.20221083	0.52801219	0.3008154	-0.1057345	0.1786331
-0.1956922	0.04358726	-0.2207458	-0.0267039	-0.2679475	-0.0287029	-0.1360759
-0.0150902	-0.0385574	0.06190982	-0.0001732	0.08750672	0.04387513	0.01598313
0.54950283	0.02573618	0.22309543	-0.0176963	0.20599089	-0.0986568	0.32722752
-0.0347652	-0.0497552	0.03108603	0.04815726	-0.0839023	-0.0408163	0.0211392
-0.1187252	-0.0054839	0.0440125	0.01521748	-0.121471	0.00592243	0.07198949
-0.289989	0.10511076	-0.2957507	-0.0254349	-0.3117593	0.13682302	-0.2082758
-0.1549849	0.16280249	-0.1970979	-0.0127584	-0.3027089	0.17146549	-0.0933736
-0.223615	-0.0030156	-0.1575179	-0.0092887	-0.0172292	-0.1255928	-0.0352101
0.07613421	-0.1426042	0.17346879	0.05541793	0.16216764	-0.0272912	0.08490576
0.71844973	0.08172533	0.2635378	0.39085103	0.43057947	-0.1837687	0.2284763
0.24720524	0.09600098	0.19612437	-0.0512056	0.44260964	-0.0767298	0.16933558
0.68268352	-0.0177631	0.2506811	-0.0132095	0.48734995	-0.2023821	0.27744199
0.0337022	0.05391468	0.19473265	0.98656091	0.19153227	0.06375105	0.00518459
-0.1873026	0.00855645	-0.0541346	-0.0268318	-0.208287	-0.0322026	-0.0287128
0.00846046	0.07920528	0.02384128	-0.0679098	-0.1791415	0.19347327	-0.0680952
0.01395583	0.06129128	0.14028938	0.99910596	0.20594628	0.02972371	-0.0077995
0.00748653	0.06481919	0.31735647	0.01544716	0.04594787	0.13657536	0.08047511
0.01366705	0.04674271	0.13521315	0.96765219	0.22162568	0.03956784	9.03E-07
0.53954883	0.04995552	0.3531784	0.03995982	0.46069829	-0.1042891	0.306813
-0.1261	-0.015004	-0.1461062	-0.0233392	-0.0905132	-0.012883	-0.0631199
0.21450197	-0.0510975	0.52534674	0.062287	0.13171889	0.02219314	0.06910201
0.65133929	0.093516	0.00962408	-0.0115663	0.45474735	-0.083448	0.27763672
-0.2174037	0.08902117	-0.1892337	-0.0216687	-0.0919671	-0.0214508	-0.0908414
0.28962123	-0.0345193	0.31145879	0.01275631	0.34027946	-0.0180998	0.05860721
-0.0348827	0.0013536	0.10048161	0.00173303	0.00121924	0.02045014	8.10E-05
0.16455146	0.01822489	0.04722331	-0.0273297	0.20405815	0.03500301	0.07467562
0.64953932	0.05226834	0.03559337	0.01066711	0.36266306	-0.0973774	0.21380164
-0.1321954	0.03064386	0.0324566	-0.0203106	-0.1631468	-0.1025802	0.00923661
0.0068862	0.05623086	0.10690836	-0.0060946	-0.0223296	0.01227746	0.04181926
0.67637067	0.02975507	0.2377076	0.16924157	0.6217799	-0.2986622	0.34523448
0.29577352	0.06650067	0.1264316	-0.005855	0.26345905	-0.0703016	0.14989182
-0.0175212	0.05410482	0.0836212	-0.0120443	-0.0759369	0.02746414	0.02949899
0.76720645	0.01866784	0.27921219	0.0521992	0.42900532	-0.1953563	0.26443219
0.23275131	-0.095219	0.35078232	-0.0243473	0.50788166	-0.2564451	0.13555021

-0.0078992	0.06152954	-0.2499051	-0.0380459	0.04996833	0.0575878	-0.0640397
-0.1201968	-0.0892677	0.12900217	-0.0132674	-0.3107728	0.24770968	-0.0379032
0.10545623	-0.0742803	0.23439483	0.12326744	0.20361193	0.14880481	0.07789183
0.01181363	0.05891754	0.13847034	0.9988656	0.20893658	0.03000982	-0.0075441
-0.1959956	0.20763551	-0.1436344	-0.0381779	-0.3575296	0.10958628	-0.1450595
-0.1399788	0.05560695	-0.1609987	-0.0520115	-0.2639963	0.10476412	-0.1119885
0.02051375	0.06465755	0.14308202	0.99853471	0.21011085	0.02459473	-0.0029512
0.08164043	0.07602151	0.09719062	0.01021319	0.05533697	0.09590678	-0.0017917
-0.059727	0.02123318	0.14557578	0.00034537	-0.0438928	0.08725339	0.09004534
0.31565139	-0.1429075	0.150822	-0.0171478	0.44046366	-0.29853	0.21509598
0.5766677	0.12128952	-0.0374052	-0.0048018	0.55915724	-0.1199627	0.19954918
1	0.13496636	0.2246012	0.01850407	0.41048023	-0.1685802	0.2606992
0.13496636	1	-0.1253905	0.05990919	-0.0638082	0.24124587	-0.0278019
0.2246012	-0.1253905	1	0.14129581	0.11274814	0.00427748	0.10992002
0.01850407	0.05990919	0.14129581	1	0.210486	0.02757503	-0.0040742
0.41048023	-0.0638082	0.11274814	0.210486	1	-0.2852903	0.1983968
-0.1685802	0.24124587	0.00427748	0.02757503	-0.2852903	1	-0.1235887
0.2606992	-0.0278019	0.10992002	-0.0040742	0.1983968	-0.1235887	1
0.63408932	-0.0229239	0.18543972	-0.0169291	0.71853422	-0.2729449	0.21287407
0.06383294	-0.0800218	-0.0664082	-0.0354931	0.17365614	0.04436831	0.04218693
0.41147279	0.0068587	0.30384797	0.00654754	0.2380886	0.02543528	0.15493177
-0.1121062	0.12103886	-0.1316351	0.23922861	-0.0689779	0.0385375	-0.0992111
0.62879877	0.03854584	0.06330937	0.02663953	0.32502765	-0.1001014	0.10736698
0.12283036	0.07813103	0.1400269	-0.0333582	0.18494039	-0.1258991	0.04767003
-0.0606208	0.1870369	-0.2811763	-0.0531084	0.04600714	0.10417231	-0.1447195
0.24586534	-0.0674731	0.43225616	0.07793374	0.40190162	0.00407411	0.06145598
0.10343975	0.01852742	-0.020685	-0.0113483	0.02912059	0.09048701	0.00624055
0.42883524	0.0303269	0.10712412	-0.0229431	0.61161892	-0.126394	0.14604268

ACADM	ACADL	AQP7	FADS2	CYP4A22	LPL	ILK
-0.3678096	0.11968302	-0.1511559	0.22196418	-0.0478059	-0.1864044	-0.0561946
-0.0673837	-0.0602532	-0.0486845	-0.0125808	-0.0347657	-0.0249779	-0.0740632
0.69224946	-0.0210207	0.424281	-0.1046031	0.61074204	0.06533355	-0.0916335
0.05023034	0.31090983	0.23069532	-0.1588155	0.06959438	0.15301874	0.06781931
0.67474987	-0.0155974	0.39712027	-0.1068174	0.54206067	0.08174488	-0.0512705
-0.0046794	0.40947356	0.14655603	0.13959196	0.17625491	-0.1144486	0.28144583
0.07719417	0.38572463	0.18351501	0.02165728	0.27441249	-0.0347746	0.15852175
-0.1050449	0.08326962	-0.2091088	0.14628734	-0.1610945	-0.0528517	0.21064747
-0.0218584	0.05433059	-0.1224662	-0.0014839	-0.0513565	-0.182184	0.02793373
0.26440035	-0.114645	0.054687	0.15925977	0.22815961	-0.1092552	-0.1352696
-0.3259923	-0.1976467	-0.2096215	0.13537348	-0.1508233	0.00289436	-0.0255811
0.00343876	-0.0414237	-0.0241348	-0.0229897	-0.0270785	-0.0246191	-0.0843932
0.50130565	0.05156682	0.32316909	-0.0162169	0.41740453	0.04712412	-0.227708
-0.0572382	0.01079019	-0.0208671	-0.0201937	-0.0298364	-0.0467312	-0.0825127
-0.1078486	-0.2954645	-0.1640245	0.03214053	-0.1639779	-0.0567313	-0.0712062
-0.3819734	0.07764847	-0.2153518	0.19305169	-0.1457882	-0.1601486	0.09350692
-0.2623973	0.07704141	-0.0924024	0.15353886	-0.0712555	-0.2024646	0.08590147
-0.1351458	-0.085304	-0.2618909	-0.0330084	-0.1955073	-0.1424814	0.08055969
0.14099462	-0.1076926	0.18775573	-0.0393698	0.02847672	-0.0865682	-0.1917837
0.63346184	-0.1041191	0.3510399	-0.0437291	0.5962217	0.04361083	-0.158682
0.5177654	0.22153392	0.19992519	-0.1306676	0.1226438	0.18942755	0.20479871
0.71778759	0.24702118	0.4784595	-0.1534821	0.5532881	0.05477481	-0.0698671
-0.0089324	-0.0476292	0.03536389	0.22746567	0.03209363	-0.0330385	-0.0844123
-0.2374463	-0.090013	-0.1527615	0.09483721	-0.1441352	-0.2359198	-0.2740769
-0.0008894	0.19784703	0.20236609	-0.1231777	0.07860151	0.12970406	0.34880556
-0.0217507	-0.0369347	0.00551434	0.24082728	0.0219847	-0.0345059	-0.050118
0.01599964	-0.0347974	0.22703706	-0.0085154	0.01458283	0.22227859	-0.0546927
-0.0018972	-0.004639	0.00370952	0.23772405	0.04018466	-0.0338204	-0.0354243
0.67706036	0.22204614	0.44576262	-0.1374748	0.37672357	0.28734322	0.05991324
-0.1431859	0.03538034	-0.1356263	0.11182476	-0.0709048	-0.0942233	0.0564761
0.22997587	-0.1714476	0.26067661	-0.0906871	0.08977055	0.16081232	-0.2043861
0.61623085	0.32518065	0.41325381	-0.1272499	0.54390131	0.07356572	0.18509192
-0.2047406	0.02531975	-0.2009649	0.27999836	-0.177295	-0.1195785	0.03796403
0.485364	0.35218742	0.38412159	-0.1177437	0.31005542	0.26896304	0.17146797
-0.0185721	0.01849474	0.04537911	-0.0151302	-0.0586156	-0.0557412	-0.0649715
0.29761091	-0.1621596	0.02412608	-0.1694496	0.06658627	0.20380141	0.0391573
0.5505479	0.31752455	0.49973133	-0.0431632	0.88719294	0.06002319	0.07216848
-0.2343954	-0.3373696	-0.2094058	0.06961925	-0.1664092	-0.1818529	-0.19634
-0.0023447	-0.0524908	0.13257221	-0.013479	-0.00873	0.32587012	0.00555869
0.76458334	0.11264434	0.40554421	-0.1063294	0.51979635	0.19175105	0.00844351
0.34691294	0.02950895	0.1916426	-0.0810452	0.2203186	0.18134411	-0.0572345
-0.0428996	0.01110942	0.13138964	0.00843577	0.00251974	0.26877896	0.01111383
0.72039867	-0.0217333	0.46014167	-0.1715268	0.66853704	0.08572246	-0.1205343
0.47853778	0.03352496	0.14334965	-0.1382123	0.07730379	0.29765122	-0.0383946

-0.0471519	0.33440362	0.07036832	0.06438313	0.18446304	0.23425694	0.28826488
-0.2225281	0.30464849	0.21075167	-0.0293731	0.03790264	-0.1159556	-0.1158113
0.17156442	0.03668691	0.22158179	-0.0292567	0.11009904	0.01115419	-0.141113
-0.0195862	-0.0300267	0.00479075	0.24000663	0.023741	-0.0317586	-0.0457966
-0.3344317	-0.1797436	-0.1851555	0.26438596	-0.1473814	0.04272229	0.12527699
-0.2538107	-0.0048485	-0.1541421	0.02736082	-0.0648637	-0.0764386	0.16674878
-0.0165818	-0.0368379	0.00872835	0.23780926	0.02599065	-0.036235	-0.05478
0.07555277	0.15482666	0.15310072	0.02947657	0.13477273	0.17338624	0.11831735
-0.0281616	-0.0325967	0.12335475	0.00232306	-0.0740577	0.11766454	-0.0359966
0.41041466	-0.2310794	0.02408734	-0.1530882	0.12525377	0.09886822	-0.0925794
0.67418523	0.40572435	0.39963487	-0.0728913	0.55753561	0.0491318	0.20142265
0.63408932	0.06383294	0.41147279	-0.1121062	0.62879877	0.12283036	-0.0606208
-0.0229239	-0.0800218	0.0068587	0.12103886	0.03854584	0.07813103	0.1870369
0.18543972	-0.0664082	0.30384797	-0.1316351	0.06330937	0.1400269	-0.2811763
-0.0169291	-0.0354931	0.00654754	0.23922861	0.02663953	-0.0333582	-0.0531084
0.71853422	0.17365614	0.2380886	-0.0689779	0.32502765	0.18494039	0.04600714
-0.2729449	0.04436831	0.02543528	0.0385375	-0.1001014	-0.1258991	0.10417231
0.21287407	0.04218693	0.15493177	-0.0992111	0.10736698	0.04767003	-0.1447195
1	0.17017384	0.39729278	-0.1668129	0.52976444	0.24657307	0.07047838
0.17017384	1	0.28619177	0.01260482	0.24484025	-0.0015658	0.1865105
0.39729278	0.28619177	1	-0.1538435	0.47386294	0.16081	-0.0312868
-0.1668129	0.01260482	-0.1538435	1	-0.0478349	-0.080793	0.1274239
0.52976444	0.24484025	0.47386294	-0.0478349	1	0.07750728	0.01564244
0.24657307	-0.0015658	0.16081	-0.080793	0.07750728	1	0.06805735
0.07047838	0.1865105	-0.0312868	0.1274239	0.01564244	0.06805735	1
0.45740948	0.23539931	0.41838167	-0.103177	0.25868625	0.2630324	0.05287789
0.001899	0.13393921	0.21544261	0.01734136	0.24326568	0.21244994	0.12706378
0.70332715	0.29403611	0.42038042	-0.1104151	0.47911586	0.15785968	0.22793756

ACSL1	FABP4	CPT2
-0.2373994	0.07373104	-0.1925362
-0.0255984	-0.0185605	-0.0765418
0.21819622	-0.0184029	0.42385972
0.43924522	0.29179908	0.08487787
0.26003481	-0.0112001	0.42207226
0.14871317	0.11361599	0.27728603
0.03404751	0.12540973	0.23967214
-0.105966	0.00352668	-0.1300301
-0.1313542	-0.0824305	0.01911847
0.04257302	-0.0857437	0.13835231
-0.2835496	0.04435819	-0.3445525
0.04259748	-0.0113781	-0.0174047
0.07712442	-0.0311434	0.23269867
-0.0233753	-0.0390233	-0.1058525
-0.0193893	-0.1510303	-0.0372042
-0.2491293	0.06139075	-0.2310545
-0.2427029	0.07116214	-0.1532974
-0.1870279	-0.0885006	-0.0819969
0.18789187	-0.0712047	0.15950951
0.20953387	-0.0271895	0.33572287
0.45231593	-0.0590075	0.48247585
0.35901683	-0.0226987	0.4805087
0.08966851	-0.0133703	-0.0315809
-0.2323014	-0.109239	-0.1810493
0.00047879	0.10773424	0.06889978
0.07818787	-0.0104955	-0.025591
0.16883438	0.28354831	0.11211748
0.09415509	-0.0140474	-0.0055582
0.57048128	-0.0026014	0.56017079
-0.1807074	0.06651062	-0.1117458
0.41388019	-0.0250409	0.20527716
0.22928551	0.14174933	0.56627954
-0.0977896	-0.0041664	-0.1336247
0.65885275	0.10024328	0.52924282
0.13194349	-0.0299871	0.00473652
0.02052856	-0.0397415	0.1531301
0.26461707	0.22348275	0.54355685
-0.1866841	-0.113996	-0.2156747
-0.0181162	0.38854469	-0.0227311
0.4367247	0.01133931	0.61099157
0.22214723	0.06397187	0.28798888
-0.039778	0.37583318	-0.0462051
0.2856845	-0.0322775	0.49414186
0.5031355	-0.0366102	0.26882365

-0.0132591	0.65524616	0.08189415
0.00801071	0.08272059	-0.1906475
0.32144621	0.02104774	0.3052228
0.07864117	-0.0099861	-0.0241544
-0.3304056	0.0574506	-0.3296255
-0.3001591	0.07717672	-0.1882206
0.07811836	-0.0119976	-0.022693
0.06065779	0.31796569	0.11578186
0.0719102	0.15714991	-0.0516201
0.14740446	-0.0755434	0.12498809
0.32407936	0.07541781	0.69091397
0.24586534	0.10343975	0.42883524
-0.0674731	0.01852742	0.0303269
0.43225616	-0.020685	0.10712412
0.07793374	-0.0113483	-0.0229431
0.40190162	0.02912059	0.61161892
0.00407411	0.09048701	-0.126394
0.06145598	0.00624055	0.14604268
0.45740948	0.001899	0.70332715
0.23539931	0.13393921	0.29403611
0.41838167	0.21544261	0.42038042
-0.103177	0.01734136	-0.1104151
0.25868625	0.24326568	0.47911586
0.2630324	0.21244994	0.15785968
0.05287789	0.12706378	0.22793756
1	0.07095361	0.50299069
0.07095361	1	0.11610326
0.50299069	0.11610326	1

Table S2: P-value for co-expression analysis

	ANGPTL4	MMP1	PCK2	PPARG	ACOX2	PLIN2
ANGPTL4	0	0.64905899	1.54E-10	0.04503304	1.22E-10	3.92E-23
MMP1	0.64905899	0	0.47681538	0.43536718	0.3810457	0.86061004
PCK2	1.54E-10	0.47681538	0	0.04775047	1.19E-225	0.0128567
PPARG	0.04503304	0.43536718	0.04775047	0	0.12011248	0.46651978
ACOX2	1.22E-10	0.3810457	1.19E-225	0.12011248	0	0.01985852
PLIN2	3.92E-23	0.86061004	0.0128567	0.46651978	0.01985852	0
CYP27A1	0.00308358	0.75160419	7.89E-07	0.04593822	0.00137871	1.49E-10
SORBS1	0.00351969	0.50916463	5.43E-06	0.87749499	0.00010703	0.26537062
OLR1	0.51529232	0.40648842	0.16987229	0.00023515	0.05848525	0.0012932
CYP8B1	0.10566515	0.40663581	2.22E-32	1.46E-05	7.25E-36	0.05749212
FABP5	4.17E-08	0.03957208	1.25E-08	0.01683138	1.55E-11	0.98674024
SLC27A6	0.00246573	0.03783888	0.92683595	0.00503572	0.94262678	0.0049828
ACSBG2	8.51E-07	0.39777025	1.28E-83	0.06279814	2.49E-73	0.00023291
GK2	0.61335319	0.98100807	0.4148775	0.29781785	0.31577399	0.45606247
ACOX3	0.69781853	0.01134635	0.02987013	1.96E-06	0.06935534	0.04832232
UBC	1.81E-35	0.47743888	2.98E-17	0.02008929	8.06E-17	9.75E-13
NR1H3	1.06E-10	0.69059858	0.00151419	0.00044707	1.39E-06	3.40E-12
ACSL5	0.55951151	0.23993307	4.05E-10	0.07676156	3.23E-10	0.03784389
DBI	0.00633266	0.65717242	2.30E-06	0.89576425	2.90E-08	0.17041
FABP1	9.58E-11	0.51825411	1.23E-175	0.01573545	2.00E-143	5.38E-05
PDPK1	2.15E-24	0.01713954	1.43E-09	0.02905854	1.04E-13	0.75176461
GK	3.79E-13	0.25093951	1.83E-119	0.0863389	3.41E-103	0.08593074
SLC27A5	0.16574353	0.76578108	0.00064892	0.72234493	6.17E-05	0.06199099
FABP6	1.51E-18	0.72184914	5.27E-05	1.31E-09	0.00019011	0.00333938
RXRB	0.27292377	0.37054848	0.62058399	0.43681705	0.3745988	0.69044882
APOA5	0.29018439	0.92242347	0.00507798	0.73766944	0.00048097	0.31389643
PLIN4	0.03719091	0.9101429	0.408924	0.00011861	0.02649444	0.44343226
APOA2	0.29295344	0.89766389	0.00571443	0.9852173	0.00042018	0.8259628
PPARA	7.84E-24	0.03574915	1.15E-54	2.57E-05	5.71E-60	0.49529093
FABP7	1.42E-05	0.79303057	0.00087478	0.2094033	0.00389594	0.23300294
SLC27A4	7.94E-22	0.52189909	7.80E-11	3.70E-06	1.66E-09	5.77E-16
SLC27A2	0.00278807	0.13837173	1.97E-77	0.06184762	1.44E-68	5.62E-05
SCD	3.90E-08	0.82214824	4.23E-08	0.00013711	3.98E-10	9.15E-13
CPT1A	3.59E-09	0.03969138	1.47E-08	1.69E-16	3.38E-10	0.07786298
ACSBG1	0.09852432	0.79693304	0.5506091	0.02928073	0.29943884	0.03298499
SCD5	1.57E-11	0.4383811	7.08E-06	0.06987882	3.68E-08	5.43E-12
CYP4A11	0.20503948	0.33613511	2.02E-72	0.0809427	1.38E-63	1.10E-08
PLTP	0.0184899	0.58004514	0.01439876	9.79E-11	0.0365797	0.00125672
ADIPOQ	0.1441429	0.85915422	0.66911043	0.70559241	0.88929031	0.33430611
ACOX1	2.51E-20	0.05962389	1.26E-106	0.14123349	1.71E-92	0.70496913
ACSL6	4.73E-09	0.37560988	4.64E-15	0.42891356	6.09E-15	0.0019962
PLIN1	0.96324183	0.79187671	0.38543461	0.92243422	0.88091059	0.65870207
ACAA1	8.15E-12	0.20977506	1.25E-170	0.4107213	5.33E-138	0.00068952
ACSL3	9.31E-36	0.1445733	2.57E-06	1.85E-10	6.49E-06	2.63E-08
CD36	1.24E-07	0.90643986	0.00012064	4.34E-14	0.00015246	2.90E-11
CPT1B	0.0115817	0.61526016	0.00117761	2.38E-05	0.00158213	0.83537703

FABP3	0.64763203	0.20805087	5.31E-05	0.12714498	1.01E-07	0.01552282
CYP7A1	0.30109684	0.9446308	0.00619686	0.76392435	0.00055996	0.37177073
CPT1C	0.01364262	0.81871484	6.57E-08	0.00056621	1.50E-08	0.02640205
PPARD	4.16E-13	0.55062707	1.94E-06	0.20705303	1.02E-05	0.81868422
APOC3	0.27427761	0.91584845	0.00269656	0.67107799	0.00025601	0.33473181
RXRG	0.02328419	0.3715872	0.17277439	0.00012438	0.03134379	0.41470581
UCP1	0.01070604	0.6619359	0.24718379	3.75E-05	0.52168869	0.02466742
ACSL4	3.23E-24	0.90211834	9.94E-16	0.05459916	4.71E-16	4.40E-12
EHHADH	0.00933033	0.12093489	1.00E-56	0.60758864	2.78E-55	8.85E-19
PCK1	1.05E-08	0.32727523	6.10E-143	0.50318088	2.13E-105	0.1211286
RXRA	0.23481043	0.13874796	0.00745659	8.86E-05	0.00177079	0.00478724
PLIN5	4.62E-12	0.85088092	2.57E-07	6.94E-11	8.20E-09	1.63E-14
APOA1	0.28533277	0.91346471	0.00318334	0.71902565	0.00027945	0.34125433
SCP2	2.46E-14	0.22775966	6.40E-29	0.03744441	1.08E-27	0.29126998
SLC27A1	0.00820352	0.36496548	4.08E-05	0.00384946	0.05202637	0.79206413
FABP2	0.00011166	0.97783367	5.69E-12	0.46624645	7.48E-16	0.02033764
ACADM	5.22E-21	0.09609275	2.47E-88	0.21502922	2.20E-82	0.90810379
ACADL	0.00304683	0.13683898	0.60404603	3.68E-15	0.70040279	4.21E-26
AQP7	0.00017666	0.22949797	4.29E-28	7.99E-09	1.62E-24	0.00027813
FADS2	2.94E-08	0.75629238	0.00966949	8.06E-05	0.00822932	0.00053941
CYP4A22	0.23802345	0.39097326	9.75E-64	0.08564634	5.84E-48	1.18E-05
LPL	3.50E-06	0.53773249	0.10666657	0.00014645	0.04339921	0.00461845
ILK	0.16535241	0.06732621	0.02350262	0.09395746	0.20566982	1.38E-12
ACSL1	2.83E-09	0.52767467	5.09E-08	3.29E-30	6.71E-11	0.00022517
FABP4	0.06856707	0.64703443	0.64983119	1.86E-13	0.78232414	0.00492704
CPT2	1.63E-06	0.05863876	4.91E-28	0.03594612	8.63E-28	3.01E-12

CYP27A1	SORBS1	OLR1	CYP8B1	FABP5	SLC27A6	ACSBG2
0.00308358	0.00351969	0.51529232	0.10566515	4.17E-08	0.00246573	8.51E-07
0.75160419	0.50916463	0.40648842	0.40663581	0.03957208	0.03783888	0.39777025
7.89E-07	5.43E-06	0.16987229	2.22E-32	1.25E-08	0.92683595	1.28E-83
0.04593822	0.87749499	0.00023515	1.46E-05	0.01683138	0.00503572	0.06279814
0.00137871	0.00010703	0.05848525	7.25E-36	1.55E-11	0.94262678	2.49E-73
1.49E-10	0.26537062	0.0012932	0.05749212	0.98674024	0.0049828	0.00023291
0	0.07034207	0.0335301	0.92681604	0.4309064	0.08762837	0.02340987
0.07034207	0	0.48524475	0.56952822	0.68649474	0.30217889	0.00795519
0.0335301	0.48524475	0	0.32377037	0.71935845	0.40395575	0.56448292
0.92681604	0.56952822	0.32377037	0	2.28E-05	0.63800325	1.26E-14
0.4309064	0.68649474	0.71935845	2.28E-05	0	0.77981161	3.77E-08
0.08762837	0.30217889	0.40395575	0.63800325	0.77981161	0	0.59392787
0.02340987	0.00795519	0.56448292	1.26E-14	3.77E-08	0.59392787	0
0.14845193	0.77182362	0.36245807	0.99541335	0.99102789	0.61087379	3.04E-05
4.62E-06	0.1955846	0.03161006	0.00520835	0.12967029	0.26665696	0.18852144
0.320512	0.00211363	0.02201311	0.0017089	6.83E-05	0.02719157	3.68E-08
1.45E-14	0.06933396	0.00017035	0.74584616	4.97E-05	0.00697901	0.04239292
3.03E-09	0.00053908	5.62E-07	0.12339394	0.92148502	0.23325727	7.75E-06
0.00119786	1.80E-10	0.21851025	0.00020643	0.70793165	0.07153212	0.13537399
0.01361151	2.82E-05	0.34736098	5.68E-50	3.34E-07	0.63208639	4.77E-57
0.14780841	0.00068551	0.97998735	0.00127728	7.30E-20	0.8048511	1.86E-05
5.79E-09	0.00016224	0.01666489	3.87E-14	3.39E-11	0.86294179	9.79E-58
0.10956547	0.12438083	0.07293372	2.97E-45	0.26587794	0.9719131	0.4401791
0.04290807	0.17991994	0.18460511	4.77E-05	0.01343051	0.14686515	0.12175368
0.15197428	0.34057715	0.00282302	1.95E-05	0.24468837	0.02811188	0.30487081
0.05666574	0.52435622	0.35157688	2.45E-44	0.5012414	0.9564093	0.61187806
0.72972059	0.82984906	0.36893219	0.13611831	0.12526172	0.00424978	0.19475822
0.01693931	0.35557098	0.27111597	9.16E-44	0.69598355	0.99089219	0.78893212
0.20154074	0.79429802	0.24987844	5.83E-11	1.51E-24	0.54361959	1.40E-34
0.16540316	0.01560819	0.81636889	0.25497581	0.01807171	0.29951793	0.08224746
0.0136185	1.26E-13	5.43E-09	0.03156658	0.02553318	0.00011202	8.61E-06
4.40E-32	0.02854636	0.40331309	2.94E-10	1.82E-10	0.10255952	9.77E-26
0.29758334	3.18E-13	0.03658685	0.21851384	0.00010635	0.09620034	0.00154059
0.35596111	0.45116392	0.00036501	0.71874562	4.31E-15	0.59797255	0.00040851
0.44158368	0.77528489	0.14556799	0.49202933	0.10298309	0.42381563	0.23353672
0.00334511	0.08239538	0.37993276	0.12025218	3.75E-09	0.9520753	0.00042356
6.34E-18	0.00017492	0.2719042	9.74E-10	3.63E-06	0.27471226	8.69E-33
0.00034293	0.61142263	0.05381587	2.32E-07	2.03E-06	0.34648426	0.52838982
0.55762581	0.09959344	0.67317853	0.98307598	0.75828291	0.19996095	0.71948081
0.00028908	0.02513588	0.56788356	4.36E-25	7.28E-17	0.76023058	5.90E-43
0.6642421	0.00082724	0.01532539	0.01888055	2.60E-06	0.07715772	2.42E-13
0.87671737	0.06073649	0.96292377	0.79545648	0.87803408	0.28092883	0.19088502
0.00222818	3.12E-08	0.28849995	4.49E-23	1.33E-12	0.70670285	3.06E-66
6.40E-08	0.96625845	0.00067956	0.68115067	1.81E-05	2.63E-07	0.00015554
3.17E-06	0.00016765	0.00409143	1.04E-08	0.00037603	0.04448266	9.72E-05
0.70922752	0.24369032	0.43604291	0.00172651	0.10730094	0.8225882	0.1276052

0.81453339	0.00012593	0.40101317	3.33E-07	0.00072	0.13289775	0.28774737
0.05026432	0.53671471	0.32456885	1.54E-44	0.54579267	0.95546718	0.56833483
0.00431217	0.07100254	1.57E-06	2.39E-05	9.50E-26	0.60154559	0.22121159
0.98595827	1.82E-08	0.37881575	0.00052095	2.70E-05	0.04823732	0.00454914
0.05171825	0.51482878	0.33893797	2.57E-45	0.46702054	0.9539087	0.68592166
0.01393586	0.04549558	0.00534261	0.40065247	0.00440726	0.54101608	0.00067217
0.04637136	0.73074464	0.12995979	0.3973914	0.79193737	0.00183177	0.00185759
1.78E-08	0.5634169	0.80345286	2.53E-06	8.35E-06	8.18E-11	7.90E-06
4.56E-16	0.35361761	0.05655378	8.04E-11	6.53E-14	0.12209739	5.51E-23
3.01E-05	0.0017499	0.07699909	3.19E-16	1.09E-06	0.70969741	1.70E-49
0.55985938	0.02932799	4.32E-08	0.21222507	0.28205571	0.34136071	0.52545452
0.00021594	0.0098008	0.00023181	4.65E-07	3.52E-08	0.12635234	2.49E-08
0.05050194	0.47594514	0.3504761	3.66E-45	0.50999608	0.99659037	0.66242913
0.1132237	0.8768577	0.9812173	3.03E-14	1.66E-11	0.03056056	2.80E-07
0.11511688	0.75627168	0.19930004	0.00890778	0.47883219	0.27888648	0.01470355
0.83768264	0.57888869	0.27690455	8.90E-06	0.00074518	0.69336408	1.03E-16
0.05651329	0.00936543	0.58970543	3.12E-11	1.36E-16	0.93239896	3.41E-40
4.13E-23	0.03962348	0.17985333	0.00454825	8.47E-07	0.30665334	0.20305819
4.98E-06	1.83E-07	0.00242623	0.17701016	1.70E-07	0.55154937	2.55E-16
0.59313395	0.00028549	0.9707995	7.69E-05	0.00079416	0.57059196	0.68911111
5.12E-12	6.33E-05	0.2049095	1.17E-08	0.00018263	0.50407728	3.71E-27
0.39085254	0.19201276	5.84E-06	0.00686795	0.94308132	0.54359215	0.2447912
8.31E-05	1.48E-07	0.49069869	0.00080166	0.52795301	0.03702257	1.26E-08
0.40084071	0.00875871	0.0011366	0.29341525	9.22E-13	0.2931377	0.05673742
0.00189732	0.93067484	0.04166542	0.03408952	0.27362281	0.7789546	0.44223424
1.98E-09	0.00127636	0.637174	0.00060502	1.80E-18	0.66765586	5.88E-09

GK2	ACOX3	UBC	NR1H3	ACSL5	DBI	FABP1
0.61335319	0.69781853	1.81E-35	1.06E-10	0.55951151	0.00633266	9.58E-11
0.98100807	0.01134635	0.47743888	0.69059858	0.23993307	0.65717242	0.51825411
0.4148775	0.02987013	2.98E-17	0.00151419	4.05E-10	2.30E-06	1.23E-175
0.29781785	1.96E-06	0.02008929	0.00044707	0.07676156	0.89576425	0.01573545
0.31577399	0.06935534	8.06E-17	1.39E-06	3.23E-10	2.90E-08	2.00E-143
0.45606247	0.04832232	9.75E-13	3.40E-12	0.03784389	0.17041	5.38E-05
0.14845193	4.62E-06	0.320512	1.45E-14	3.03E-09	0.00119786	0.01361151
0.77182362	0.1955846	0.00211363	0.06933396	0.00053908	1.80E-10	2.82E-05
0.36245807	0.03161006	0.02201311	0.00017035	5.62E-07	0.21851025	0.34736098
0.99541335	0.00520835	0.0017089	0.74584616	0.12339394	0.00020643	5.68E-50
0.99102789	0.12967029	6.83E-05	4.97E-05	0.92148502	0.70793165	3.34E-07
0.61087379	0.26665696	0.02719157	0.00697901	0.23325727	0.07153212	0.63208639
3.04E-05	0.18852144	3.68E-08	0.04239292	7.75E-06	0.13537399	4.77E-57
0	0.99611147	0.53643388	0.91461044	0.24663639	0.72611275	0.81162009
0.99611147	0	0.95173872	0.15428788	2.84E-07	0.03492777	0.32637814
0.53643388	0.95173872	0	1.87E-11	0.01919099	0.00030988	2.48E-16
0.91461044	0.15428788	1.87E-11	0	0.0156671	0.20524073	8.78E-05
0.24663639	2.84E-07	0.01919099	0.0156671	0	0.00012554	5.18E-06
0.72611275	0.03492777	0.00030988	0.20524073	0.00012554	0	3.26E-06
0.81162009	0.32637814	2.48E-16	8.78E-05	5.18E-06	3.26E-06	0
0.05166273	0.40381759	9.42E-14	2.02E-10	0.11819181	0.03373134	7.62E-05
0.77721057	0.00188018	3.11E-17	0.00028487	2.18E-06	8.27E-10	4.39E-79
0.12486976	0.54305055	0.21988135	0.89420943	0.28005991	0.06039053	2.87E-25
0.30275072	3.33E-06	2.00E-07	3.73E-16	0.03942229	2.87E-06	0.00117836
0.875638	0.0080959	0.76652293	0.36725486	0.17102519	3.32E-05	0.18044669
0.23019505	0.67824586	0.57399473	0.76029577	0.84177527	0.18907226	8.24E-23
0.91359775	0.87672464	0.00333686	0.3303063	0.13176697	1.61E-15	0.37355717
0.24316165	0.93711036	0.57950889	0.72606997	0.85484838	0.09503848	1.24E-21
0.16007246	0.83270373	1.32E-27	6.33E-10	0.0003352	0.13479889	2.51E-37
0.58178635	0.89785645	0.00413848	0.00047039	0.68996068	0.81405531	0.00563633
0.78157251	0.0409124	5.79E-20	6.69E-18	4.40E-09	1.53E-14	1.05E-10
0.07068802	1.17E-09	1.62E-07	0.06233517	6.09E-07	0.11525385	7.24E-35
0.14593594	0.651892	2.06E-09	1.19E-27	0.10522592	0.58252039	5.84E-09
0.45096109	0.13768221	1.51E-07	2.41E-13	0.03078544	0.02795153	9.34E-07
0.56619621	0.99920019	0.08649248	0.03821723	0.5323024	0.0779519	0.48940511
0.09697645	0.14340787	2.00E-05	3.82E-24	0.40832637	0.46318199	5.61E-06
0.45500777	1.27E-05	3.25E-05	0.22953677	7.40E-08	0.96978326	1.42E-49
0.47314526	1.09E-22	0.03461425	0.00015342	0.02302139	1.00E-07	0.10177455
0.67151849	0.40431807	0.36873436	0.95959596	0.98809273	0.94866529	0.96292831
0.40373213	0.84429537	3.25E-21	3.61E-10	0.0176423	2.45E-06	1.22E-81
0.85504077	0.74050996	9.59E-05	5.69E-07	0.00954433	0.01389318	1.87E-10
0.71496588	0.74371008	0.40023678	0.17407963	0.93580291	0.92804719	0.50797786
0.34123958	0.36311932	1.09E-19	1.09E-06	5.04E-07	4.50E-09	5.45E-170
0.46731698	0.61620961	1.26E-13	2.06E-33	0.69736912	0.15690334	7.76E-07
0.25137264	1.50E-10	7.55E-07	0.05072362	0.37924523	1.41E-12	6.84E-06
0.00422957	0.03142232	0.0768489	0.00015735	0.58611903	0.01210484	0.00242071

0.62492022	0.27813266	0.00016218	0.87261149	2.59E-06	5.47E-15	8.01E-06
0.22514922	0.71426161	0.60639387	0.73818787	0.86900161	0.20921229	1.84E-22
0.06249926	0.68470317	0.01293738	0.29886363	0.89732922	1.13E-05	6.67E-07
0.84387716	0.00577474	1.83E-07	0.89249464	0.05624208	3.71E-08	1.12E-05
0.24296713	0.6540555	0.51221864	0.80094124	0.85519703	0.14653379	1.06E-23
0.14279617	6.23E-06	0.03213478	0.71987016	0.43818979	0.08396069	0.33782385
0.31529514	0.95018784	0.0427451	0.08952895	0.98404007	0.89977015	0.36761414
0.64490877	0.81015181	4.13E-21	1.19E-24	0.10942889	0.02888067	8.32E-18
0.00637502	2.72E-05	5.24E-05	0.16328003	0.08721499	0.12600687	5.89E-29
0.39098092	0.00329177	2.65E-13	0.00011986	2.31E-08	0.05999998	4.38E-98
0.21940496	0.89239297	0.00932084	5.28E-05	0.94070061	0.00040652	0.04344953
0.44307598	0.27738259	8.45E-14	9.10E-07	9.23E-05	1.62E-05	3.63E-11
0.23458793	0.70736038	0.53031541	0.75296321	0.81876202	0.171284	9.79E-24
0.03814051	0.00263349	3.07E-15	2.05E-14	0.67080895	5.65E-05	5.69E-29
0.31380614	0.88384665	0.00069617	2.03E-05	0.00186821	0.50073108	4.83E-06
0.60200746	0.07538257	2.05E-07	0.02097879	0.38494234	0.03588499	1.12E-08
0.15762683	0.00762646	1.17E-22	4.44E-11	0.00081067	0.00047317	7.46E-70
0.79010219	8.95E-14	0.05507123	0.05700517	0.03502193	0.00771504	0.01001269
0.60669154	4.63E-05	7.64E-08	0.02235664	4.85E-11	2.97E-06	3.70E-19
0.61835413	0.42775401	1.53E-06	0.00013892	0.41537755	0.33127744	0.28049081
0.46163185	4.65E-05	0.00029964	0.07841555	1.12E-06	0.48230476	4.42E-60
0.2487516	0.16134448	7.00E-05	4.49E-07	0.00041128	0.03239809	0.28179524
0.04146152	0.07862302	0.02079552	0.0337601	0.04653961	1.79E-06	8.17E-05
0.56414486	0.63241026	4.26E-10	1.22E-09	3.24E-06	2.92E-06	1.72E-07
0.33555513	0.00017889	0.12956882	0.07880842	0.02871273	0.07862899	0.50232988
0.00883153	0.35858604	7.56E-09	0.00014237	0.04275506	7.49E-05	1.46E-17

PDPK1	GK	SLC27A5	FABP6	RXRB	APOA5	PLIN4
2.15E-24	3.79E-13	0.16574353	1.51E-18	0.27292377	0.29018439	0.03719091
0.01713954	0.25093951	0.76578108	0.72184914	0.37054848	0.92242347	0.9101429
1.43E-09	1.83E-119	0.00064892	5.27E-05	0.62058399	0.00507798	0.408924
0.02905854	0.0863389	0.72234493	1.31E-09	0.43681705	0.73766944	0.00011861
1.04E-13	3.41E-103	6.17E-05	0.00019011	0.3745988	0.00048097	0.02649444
0.75176461	0.08593074	0.06199099	0.00333938	0.69044882	0.31389643	0.44343226
0.14780841	5.79E-09	0.10956547	0.04290807	0.15197428	0.05666574	0.72972059
0.00068551	0.00016224	0.12438083	0.17991994	0.34057715	0.52435622	0.82984906
0.97998735	0.01666489	0.07293372	0.18460511	0.00282302	0.35157688	0.36893219
0.00127728	3.87E-14	2.97E-45	4.77E-05	1.95E-05	2.45E-44	0.13611831
7.30E-20	3.39E-11	0.26587794	0.01343051	0.24468837	0.5012414	0.12526172
0.8048511	0.86294179	0.9719131	0.14686515	0.02811188	0.9564093	0.00424978
1.86E-05	9.79E-58	0.4401791	0.12175368	0.30487081	0.61187806	0.19475822
0.05166273	0.77721057	0.12486976	0.30275072	0.875638	0.23019505	0.91359775
0.40381759	0.00188018	0.54305055	3.33E-06	0.0080959	0.67824586	0.87672464
9.42E-14	3.11E-17	0.21988135	2.00E-07	0.76652293	0.57399473	0.00333686
2.02E-10	0.00028487	0.89420943	3.73E-16	0.36725486	0.76029577	0.3303063
0.11819181	2.18E-06	0.28005991	0.03942229	0.17102519	0.84177527	0.13176697
0.03373134	8.27E-10	0.06039053	2.87E-06	3.32E-05	0.18907226	1.61E-15
7.62E-05	4.39E-79	2.87E-25	0.00117836	0.18044669	8.24E-23	0.37355717
0	2.99E-19	0.08493226	1.84E-11	0.01051819	0.20991022	0.29869752
2.99E-19	0	0.94451187	8.62E-05	0.26504543	0.62830621	0.02930712
0.08493226	0.94451187	0	0.83098821	0.12849985	0	0.51406666
1.84E-11	8.62E-05	0.83098821	0	1.46E-09	0.50804904	0.25407076
0.01051819	0.26504543	0.12849985	1.46E-09	0	0.09366923	0.64283333
0.20991022	0.62830621	0	0.50804904	0.09366923	0	0.70390001
0.29869752	0.02930712	0.51406666	0.25407076	0.64283333	0.70390001	0
0.19046883	0.90978226	0	0.42886903	0.21381827	0	0.6171532
1.07E-78	2.66E-66	0.25512692	1.06E-12	0.00885331	0.34536694	0.01879689
7.23E-05	0.0093876	0.28745985	0.05949821	0.51174119	0.58528463	0.14719278
0.00101357	2.76E-09	0.00184069	2.63E-05	0.12100202	0.13003074	1.40E-05
2.74E-16	1.26E-75	0.66261615	6.08E-08	0.16120387	0.72329536	0.6141474
0.14892342	5.95E-08	0.16742133	2.65E-06	2.21E-06	0.62089374	0.83692404
1.93E-46	9.26E-20	0.65967842	1.80E-16	2.25E-06	0.75250367	0.28732484
0.07335549	0.29164121	0.77643648	0.31135959	0.22356499	0.94338305	0.18073061
4.13E-07	0.03010955	0.71661971	8.61E-10	1.01E-05	0.49439272	0.43230378
1.83E-05	6.90E-60	0.72947621	6.64E-05	0.030665	0.86721509	0.58660576
0.00973556	0.00027313	0.57745923	1.22E-12	7.08E-08	0.61027869	0.00074709
0.86928264	0.67871843	0.91179909	0.3066227	0.05591344	0.88959416	3.17E-100
6.14E-38	3.36E-99	1.29E-05	1.49E-08	0.15279965	3.78E-05	0.05851233
4.13E-08	3.25E-13	0.78816694	0.0160069	0.72711422	0.84210186	0.59163156
0.56011724	0.44875109	0.83136155	0.77979999	0.00121287	0.78303811	4.78E-89
2.63E-09	3.28E-97	0.05947793	0.00048675	0.17398634	0.261917	0.11619617
6.02E-38	1.20E-14	0.70596452	6.60E-16	0.00216796	0.53913098	0.23049273
0.22725604	0.00210136	0.06698444	8.18E-05	0.00092045	0.39664926	0.80542339
0.12389967	0.68545386	0.77936285	0.87430662	3.23E-23	0.78196096	0.2659073

0.48067212	4.43E-05	0.00020305	7.84E-07	0.00028699	0.00281802	7.00E-13
0.20128705	0.65757219	0	0.48871863	0.12795678	0	0.71741085
5.85E-13	6.66E-17	0.61940878	0.30877079	1.56E-06	0.39715678	0.80763916
3.10E-06	2.81E-11	0.04337983	0.01472773	3.07E-15	0.22749694	0.2238874
0.20907609	0.72850752	0	0.51252979	0.08394507	0	0.64989397
0.67585658	0.06192917	0.40057474	2.90E-05	0.00019936	0.93843745	4.82E-16
0.54564322	0.27670325	0.51613831	0.05375374	0.19653554	0.94956667	9.45E-33
1.85E-12	6.66E-16	0.76150591	2.49E-09	0.00879859	0.61800648	0.95690578
8.53E-32	3.80E-69	0.50562648	0.00013905	0.80972086	0.86308944	0.91621691
5.85E-10	4.97E-85	0.40563739	3.14E-06	0.83467885	0.7306385	0.85348101
0.01761481	0.66123256	0.18321387	0.83283056	0.05035982	0.13019233	0.10945883
1.03E-06	3.29E-10	1.23E-06	0.18143129	0.55640107	0.00050546	9.19E-16
0.20624497	0.74452721	0	0.50797099	0.0935186	0	0.70315114
1.06E-30	9.11E-38	1.85E-06	2.05E-07	8.38E-06	2.82E-07	0.25678124
0.0580195	4.54E-07	0.11544131	0.42686123	1.45E-06	0.46332634	0.00071208
2.58E-05	2.92E-12	0.89823334	0.47867909	0.0926249	0.84742874	0.04677083
3.33E-43	7.97E-98	0.82560091	2.81E-09	0.98249536	0.59153996	0.6930634
3.14E-08	6.03E-10	0.23976524	0.02608528	8.25E-07	0.36208018	0.39054115
6.29E-07	2.81E-36	0.38286909	0.00015031	4.55E-07	0.89179944	1.39E-08
0.00120721	0.00013973	1.30E-08	0.01904179	0.0022873	1.64E-09	0.83362118
0.00239085	2.72E-50	0.42842882	0.00035133	0.05214541	0.58755696	0.71903758
2.41E-06	0.1763148	0.41495199	3.57E-09	0.00131312	0.39452626	2.81E-08
3.29E-07	0.08442371	0.03697946	5.45E-12	6.40E-19	0.21605792	0.17696505
3.81E-32	5.03E-20	0.02666474	6.25E-09	0.9905767	0.05339895	2.73E-05
0.14515661	0.57548055	0.7415279	0.00687625	0.00769133	0.79570575	9.23E-13
6.04E-37	1.29E-36	0.43584654	6.69E-06	0.08882505	0.52779445	0.00552962

APOA2	PPARA	FABP7	SLC27A4	SLC27A2	SCD	CPT1A
0.29295344	7.84E-24	1.42E-05	7.94E-22	0.00278807	3.90E-08	3.59E-09
0.89766389	0.03574915	0.79303057	0.52189909	0.13837173	0.82214824	0.03969138
0.00571443	1.15E-54	0.00087478	7.80E-11	1.97E-77	4.23E-08	1.47E-08
0.9852173	2.57E-05	0.2094033	3.70E-06	0.06184762	0.00013711	1.69E-16
0.00042018	5.71E-60	0.00389594	1.66E-09	1.44E-68	3.98E-10	3.38E-10
0.8259628	0.49529093	0.23300294	5.77E-16	5.62E-05	9.15E-13	0.07786298
0.01693931	0.20154074	0.16540316	0.0136185	4.40E-32	0.29758334	0.35596111
0.35557098	0.79429802	0.01560819	1.26E-13	0.02854636	3.18E-13	0.45116392
0.27111597	0.24987844	0.81636889	5.43E-09	0.40331309	0.03658685	0.00036501
9.16E-44	5.83E-11	0.25497581	0.03156658	2.94E-10	0.21851384	0.71874562
0.69598355	1.51E-24	0.01807171	0.02553318	1.82E-10	0.00010635	4.31E-15
0.99089219	0.54361959	0.29951793	0.00011202	0.10255952	0.09620034	0.59797255
0.78893212	1.40E-34	0.08224746	8.61E-06	9.77E-26	0.00154059	0.00040851
0.24316165	0.16007246	0.58178635	0.78157251	0.07068802	0.14593594	0.45096109
0.93711036	0.83270373	0.89785645	0.0409124	1.17E-09	0.651892	0.13768221
0.57950889	1.32E-27	0.00413848	5.79E-20	1.62E-07	2.06E-09	1.51E-07
0.72606997	6.33E-10	0.00047039	6.69E-18	0.06233517	1.19E-27	2.41E-13
0.85484838	0.0003352	0.68996068	4.40E-09	6.09E-07	0.10522592	0.03078544
0.09503848	0.13479889	0.81405531	1.53E-14	0.11525385	0.58252039	0.02795153
1.24E-21	2.51E-37	0.00563633	1.05E-10	7.24E-35	5.84E-09	9.34E-07
0.19046883	1.07E-78	7.23E-05	0.00101357	2.74E-16	0.14892342	1.93E-46
0.90978226	2.66E-66	0.0093876	2.76E-09	1.26E-75	5.95E-08	9.26E-20
0	0.25512692	0.28745985	0.00184069	0.66261615	0.16742133	0.65967842
0.42886903	1.06E-12	0.05949821	2.63E-05	6.08E-08	2.65E-06	1.80E-16
0.21381827	0.00885331	0.51174119	0.12100202	0.16120387	2.21E-06	2.25E-06
0	0.34536694	0.58528463	0.13003074	0.72329536	0.62089374	0.75250367
0.6171532	0.01879689	0.14719278	1.40E-05	0.6141474	0.83692404	0.28732484
0	0.36293134	0.44428779	0.13090718	0.87952099	0.47566015	0.62096763
0.36293134	0	1.57E-05	2.58E-11	3.92E-42	4.80E-06	6.27E-67
0.44428779	1.57E-05	0	2.86E-07	0.03416879	0.0039846	0.0003882
0.13090718	2.58E-11	2.86E-07	0	0.18047536	2.15E-20	1.12E-20
0.87952099	3.92E-42	0.03416879	0.18047536	0	0.00107073	5.13E-13
0.47566015	4.80E-06	0.0039846	2.15E-20	0.00107073	0	5.00E-09
0.62096763	6.27E-67	0.0003882	1.12E-20	5.13E-13	5.00E-09	0
0.95832515	0.01368506	0.49932521	0.19504946	0.0421951	0.90657993	0.01704989
0.42961292	5.52E-08	0.00526327	3.93E-05	0.09777991	9.83E-22	0.00028307
0.57206253	3.78E-29	0.03496538	0.03717123	6.36E-77	7.12E-06	2.57E-18
0.49007716	1.03E-07	0.86789374	0.43156847	3.68E-11	3.17E-05	1.74E-16
0.85172188	0.93990896	0.75066066	0.43131112	0.6050705	0.35908446	0.87852111
4.72E-05	7.51E-104	0.00010291	6.32E-16	1.02E-67	6.00E-08	8.53E-40
0.83480461	5.83E-15	0.11407728	7.67E-10	5.84E-10	0.00014483	9.02E-08
0.76830312	0.6005668	0.91183763	0.46383806	0.35689339	0.00882496	0.69630374
0.19622361	1.94E-48	0.00333853	1.35E-15	5.94E-48	1.55E-15	1.42E-11
0.49901056	1.14E-33	1.32E-05	3.18E-39	0.00510586	0.00029045	7.91E-44
0.30287438	0.01277839	0.0038187	1.59E-15	2.57E-06	0.0066556	0.05988469
0.78101749	0.51711558	0.73224439	0.73151175	0.00908886	0.08748797	0.03052162

0.00092597	6.05E-05	0.00021901	1.10E-07	0.18562106	0.37097007	0.00062775
0	0.35645012	0.58418435	0.15001944	0.70489786	0.56529885	0.72068723
0.39085557	2.74E-14	0.55219647	0.21130436	3.31E-08	0.01593069	6.78E-09
0.17290712	4.27E-08	0.66175585	2.64E-09	0.03202603	0.31408306	2.76E-05
0	0.2799987	0.59485492	0.12390567	0.77381072	0.61938229	0.78054612
0.04087218	0.04654143	0.2166623	0.00129541	0.00397974	0.0238798	4.26E-05
0.9224135	0.2294814	0.26925151	0.00796453	0.0203697	0.78851151	0.32446453
0.61389782	1.49E-11	2.03E-05	3.57E-19	0.00019238	1.45E-07	2.09E-05
0.99302654	1.20E-51	0.04347875	0.27980649	1.96E-101	0.73756982	1.80E-18
0.73600257	1.89E-47	0.00178967	8.62E-08	4.87E-75	5.70E-08	2.85E-13
0.24863531	0.21755246	0.71128147	0.20720529	0.02078309	0.02778377	0.39434261
0.00080575	2.18E-19	0.00029055	1.20E-44	0.81233853	2.47E-06	3.28E-15
0	0.32407684	0.56474694	0.12405431	0.77539538	0.59293857	0.75300248
3.09E-08	1.97E-33	0.02526309	0.00110067	1.63E-32	0.02299934	4.99E-18
0.32884956	0.0098909	0.75062981	0.5840193	0.03920039	0.59666416	0.65522172
0.99998223	8.75E-15	0.11909481	0.08788994	2.82E-12	0.02473598	0.14790961
0.96267309	3.81E-83	0.00038466	8.91E-09	3.61E-65	3.31E-07	1.98E-37
0.908893	2.91E-08	0.38264765	2.04E-05	1.63E-16	0.53217932	2.79E-19
0.92709044	3.65E-31	0.0007762	6.00E-11	1.33E-26	5.48E-07	6.45E-23
2.69E-09	0.00065587	0.0056548	0.02498253	0.00162269	1.81E-12	0.0035612
0.32135968	4.88E-22	0.07989953	0.02649195	2.46E-48	1.04E-05	4.42E-15
0.40399145	4.44E-13	0.0198344	6.53E-05	0.06919152	0.00307273	1.38E-11
0.38205684	0.13907172	0.16324094	3.48E-07	4.11E-06	0.34884769	2.03E-05
0.01992422	4.86E-54	6.97E-06	1.10E-26	9.90E-09	0.01560384	2.52E-77
0.72894022	0.94883495	0.10048858	0.53670741	0.00044074	0.91814012	0.0131741
0.89094356	9.13E-52	0.00568899	3.08E-07	4.20E-53	0.00092934	2.11E-45

ACSBG1	SCD5	CYP4A11	PLTP	ADIPOQ	ACOX1	ACSL6
0.09852432	1.57E-11	0.20503948	0.0184899	0.1441429	2.51E-20	4.73E-09
0.79693304	0.4383811	0.33613511	0.58004514	0.85915422	0.05962389	0.37560988
0.5506091	7.08E-06	2.02E-72	0.01439876	0.66911043	1.26E-106	4.64E-15
0.02928073	0.06987882	0.0809427	9.79E-11	0.70559241	0.14123349	0.42891356
0.29943884	3.68E-08	1.38E-63	0.0365797	0.88929031	1.71E-92	6.09E-15
0.03298499	5.43E-12	1.10E-08	0.00125672	0.33430611	0.70496913	0.0019962
0.44158368	0.00334511	6.34E-18	0.00034293	0.55762581	0.00028908	0.6642421
0.77528489	0.08239538	0.00017492	0.61142263	0.09959344	0.02513588	0.00082724
0.14556799	0.37993276	0.2719042	0.05381587	0.67317853	0.56788356	0.01532539
0.49202933	0.12025218	9.74E-10	2.32E-07	0.98307598	4.36E-25	0.01888055
0.10298309	3.75E-09	3.63E-06	2.03E-06	0.75828291	7.28E-17	2.60E-06
0.42381563	0.9520753	0.27471226	0.34648426	0.19996095	0.76023058	0.07715772
0.23353672	0.00042356	8.69E-33	0.52838982	0.71948081	5.90E-43	2.42E-13
0.56619621	0.09697645	0.45500777	0.47314526	0.67151849	0.40373213	0.85504077
0.99920019	0.14340787	1.27E-05	1.09E-22	0.40431807	0.84429537	0.74050996
0.08649248	2.00E-05	3.25E-05	0.03461425	0.36873436	3.25E-21	9.59E-05
0.03821723	3.82E-24	0.22953677	0.00015342	0.95959596	3.61E-10	5.69E-07
0.5323024	0.40832637	7.40E-08	0.02302139	0.98809273	0.0176423	0.00954433
0.0779519	0.46318199	0.96978326	1.00E-07	0.94866529	2.45E-06	0.01389318
0.48940511	5.61E-06	1.42E-49	0.10177455	0.96292831	1.22E-81	1.87E-10
0.07335549	4.13E-07	1.83E-05	0.00973556	0.86928264	6.14E-38	4.13E-08
0.29164121	0.03010955	6.90E-60	0.00027313	0.67871843	3.36E-99	3.25E-13
0.77643648	0.71661971	0.72947621	0.57745923	0.91179909	1.29E-05	0.78816694
0.31135959	8.61E-10	6.64E-05	1.22E-12	0.3066227	1.49E-08	0.0160069
0.22356499	1.01E-05	0.030665	7.08E-08	0.05591344	0.15279965	0.72711422
0.94338305	0.49439272	0.86721509	0.61027869	0.88959416	3.78E-05	0.84210186
0.18073061	0.43230378	0.58660576	0.00074709	3.17E-100	0.05851233	0.59163156
0.95832515	0.42961292	0.57206253	0.49007716	0.85172188	4.72E-05	0.83480461
0.01368506	5.52E-08	3.78E-29	1.03E-07	0.93990896	7.51E-104	5.83E-15
0.49932521	0.00526327	0.03496538	0.86789374	0.75066066	0.00010291	0.11407728
0.19504946	3.93E-05	0.03717123	0.43156847	0.43131112	6.32E-16	7.67E-10
0.0421951	0.09777991	6.36E-77	3.68E-11	0.6050705	1.02E-67	5.84E-10
0.90657993	9.83E-22	7.12E-06	3.17E-05	0.35908446	6.00E-08	0.00014483
0.01704989	0.00028307	2.57E-18	1.74E-16	0.87852111	8.53E-40	9.02E-08
0	0.0662169	0.08243472	0.06690945	0.77645913	0.89852924	0.48019398
0.0662169	0	0.18789007	6.61E-05	0.29788726	1.66E-09	6.15E-05
0.08243472	0.18789007	0	2.45E-06	0.55138264	4.92E-51	5.00E-10
0.06690945	6.61E-05	2.45E-06	0	0.59391496	0.00010258	0.01957104
0.77645913	0.29788726	0.55138264	0.59391496	0	0.85441281	0.72801059
0.89852924	1.66E-09	4.92E-51	0.00010258	0.85441281	0	5.02E-26
0.48019398	6.15E-05	5.00E-10	0.01957104	0.72801059	5.02E-26	0
0.80205304	0.85485858	0.81574792	0.2644651	1.23E-247	0.32988869	0.81937106
0.66622002	1.67E-10	2.16E-66	0.02125426	0.86686481	7.60E-84	1.11E-14
0.00052175	2.90E-09	0.09015352	0.00056448	0.61639351	8.32E-33	4.01E-14
0.21102036	0.00118006	1.08E-06	1.02E-08	0.03799926	0.01050883	0.74599749
0.07075766	2.61E-08	0.53156903	0.0049908	0.77876276	1.19E-07	0.07354222

0.05195042	0.12659404	0.00571191	0.4882055	0.78813065	9.16E-05	0.15004359
0.96919053	0.51115045	0.8417566	0.56036819	0.9016389	4.68E-05	0.86160358
0.0453087	0.20246469	1.78E-05	0.02051395	0.0328468	5.03E-15	0.00861894
0.15026061	1.78E-07	0.04833781	0.08149919	0.66282075	1.08E-11	1.02E-05
0.94736242	0.49921966	0.79303913	0.72036511	0.88619057	2.12E-05	0.8710473
0.2901659	0.26169099	0.00056319	0.00071484	1.57E-22	0.13054111	0.00026934
4.36E-08	0.76078121	0.03489938	0.78549249	1.00E-42	0.93092094	0.5043675
0.31252555	2.02E-17	0.01109386	0.46323771	0.2836137	7.55E-27	5.90E-10
0.03794743	0.10347081	2.63E-80	2.97E-07	0.17164379	4.56E-79	5.71E-10
0.38938071	4.37E-05	1.68E-74	0.0010553	0.86511289	6.44E-83	8.41E-14
0.97336331	0.65299505	0.19697193	0.44959286	0.16507919	0.46285453	0.10053962
0.0129569	0.24379829	0.37978728	0.423222	0.00817448	2.69E-09	0.00173997
0.96590077	0.50012785	0.79244136	0.61632264	0.8804951	2.61E-05	0.88515994
0.97600642	3.63E-07	1.98E-20	5.09E-05	0.58170797	1.20E-66	3.69E-11
0.61390108	0.38774627	0.01604851	0.01117679	0.76198955	4.70E-14	0.0825047
0.99840515	0.06508711	9.51E-08	0.81976151	0.3020531	1.53E-18	0.00020039
0.64682834	5.81E-14	1.03E-49	4.52E-09	0.95387703	2.83E-118	1.02E-18
0.64820058	5.65E-05	8.86E-16	9.92E-18	0.19506998	0.00531059	0.46656503
0.26272239	0.55169382	6.49E-40	1.75E-07	0.00102064	1.37E-25	1.83E-06
0.70896275	2.55E-05	0.28676857	0.08553424	0.73950164	0.00852913	0.04523083
0.14785179	0.10010149	9.63E-207	3.57E-05	0.82949221	1.38E-43	3.74E-08
0.16879633	3.76E-07	0.13834661	6.08E-06	1.40E-16	1.80E-06	6.46E-06
0.10862581	0.33389657	0.07465758	1.00E-06	0.89093454	0.83500526	0.15765331
0.00107906	0.61254231	3.00E-11	3.38E-06	0.6549296	7.60E-30	2.87E-08
0.45937198	0.32673002	2.36E-08	0.00478394	1.88E-23	0.77968862	0.11418395
0.90698674	0.0001448	2.89E-48	7.30E-08	0.57493451	8.41E-64	3.92E-13

PLIN1	ACAA1	ACSL3	CD36	CPT1B	FABP3	CYP7A1
0.96324183	8.15E-12	9.31E-36	1.24E-07	0.0115817	0.64763203	0.30109684
0.79187671	0.20977506	0.1445733	0.90643986	0.61526016	0.20805087	0.9446308
0.38543461	1.25E-170	2.57E-06	0.00012064	0.00117761	5.31E-05	0.00619686
0.92243422	0.4107213	1.85E-10	4.34E-14	2.38E-05	0.12714498	0.76392435
0.88091059	5.33E-138	6.49E-06	0.00015246	0.00158213	1.01E-07	0.00055996
0.65870207	0.00068952	2.63E-08	2.90E-11	0.83537703	0.01552282	0.37177073
0.87671737	0.00222818	6.40E-08	3.17E-06	0.70922752	0.81453339	0.05026432
0.06073649	3.12E-08	0.96625845	0.00016765	0.24369032	0.00012593	0.53671471
0.96292377	0.28849995	0.00067956	0.00409143	0.43604291	0.40101317	0.32456885
0.79545648	4.49E-23	0.68115067	1.04E-08	0.00172651	3.33E-07	1.54E-44
0.87803408	1.33E-12	1.81E-05	0.00037603	0.10730094	0.00072	0.54579267
0.28092883	0.70670285	2.63E-07	0.04448266	0.8225882	0.13289775	0.95546718
0.19088502	3.06E-66	0.00015554	9.72E-05	0.1276052	0.28774737	0.56833483
0.71496588	0.34123958	0.46731698	0.25137264	0.00422957	0.62492022	0.22514922
0.74371008	0.36311932	0.61620961	1.50E-10	0.03142232	0.27813266	0.71426161
0.40023678	1.09E-19	1.26E-13	7.55E-07	0.0768489	0.00016218	0.60639387
0.17407963	1.09E-06	2.06E-33	0.05072362	0.00015735	0.87261149	0.73818787
0.93580291	5.04E-07	0.69736912	0.37924523	0.58611903	2.59E-06	0.86900161
0.92804719	4.50E-09	0.15690334	1.41E-12	0.01210484	5.47E-15	0.20921229
0.50797786	5.45E-170	7.76E-07	6.84E-06	0.00242071	8.01E-06	1.84E-22
0.56011724	2.63E-09	6.02E-38	0.22725604	0.12389967	0.48067212	0.20128705
0.44875109	3.28E-97	1.20E-14	0.00210136	0.68545386	4.43E-05	0.65757219
0.83136155	0.05947793	0.70596452	0.06698444	0.77936285	0.00020305	0
0.77979999	0.00048675	6.60E-16	8.18E-05	0.87430662	7.84E-07	0.48871863
0.00121287	0.17398634	0.00216796	0.00092045	3.23E-23	0.00028699	0.12795678
0.78303811	0.261917	0.53913098	0.39664926	0.78196096	0.00281802	0
4.78E-89	0.11619617	0.23049273	0.80542339	0.2659073	7.00E-13	0.71741085
0.76830312	0.19622361	0.49901056	0.30287438	0.78101749	0.00092597	0
0.6005668	1.94E-48	1.14E-33	0.01277839	0.51711558	6.05E-05	0.35645012
0.91183763	0.00333853	1.32E-05	0.0038187	0.73224439	0.00021901	0.58418435
0.46383806	1.35E-15	3.18E-39	1.59E-15	0.73151175	1.10E-07	0.15001944
0.35689339	5.94E-48	0.00510586	2.57E-06	0.00908886	0.18562106	0.70489786
0.00882496	1.55E-15	0.00029045	0.0066556	0.08748797	0.37097007	0.56529885
0.69630374	1.42E-11	7.91E-44	0.05988469	0.03052162	0.00062775	0.72068723
0.80205304	0.66622002	0.00052175	0.21102036	0.07075766	0.05195042	0.96919053
0.85485858	1.67E-10	2.90E-09	0.00118006	2.61E-08	0.12659404	0.51115045
0.81574792	2.16E-66	0.09015352	1.08E-06	0.53156903	0.00571191	0.8417566
0.2644651	0.02125426	0.00056448	1.02E-08	0.0049908	0.4882055	0.56036819
1.23E-247	0.86686481	0.61639351	0.03799926	0.77876276	0.78813065	0.9016389
0.32988869	7.60E-84	8.32E-33	0.01050883	1.19E-07	9.16E-05	4.68E-05
0.81937106	1.11E-14	4.01E-14	0.74599749	0.07354222	0.15004359	0.86160358
0	0.70699738	0.54873442	0.00530206	0.18467177	0.93376428	0.79657962
0.70699738	0	6.30E-09	8.51E-08	0.02909636	1.24E-07	0.27073711
0.54873442	6.30E-09	0	0.00978576	0.00014492	0.31007326	0.52084295
0.00530206	8.51E-08	0.00978576	0	0.26870673	0.00127213	0.41836515
0.18467177	0.02909636	0.00014492	0.26870673	0	0.86255302	0.80974937

0.93376428	1.24E-07	0.31007326	0.00127213	0.86255302	0	0.00277004
0.79657962	0.27073711	0.52084295	0.41836515	0.80974937	0.00277004	0
0.00333715	2.40E-10	0.0028364	0.00031307	0.13914303	3.73E-10	0.3838213
0.28451785	2.54E-06	4.47E-12	8.01E-08	0.10607343	1.48E-05	0.22946352
0.78010169	0.18488578	0.52978783	0.3449973	0.7351112	0.00285516	0
4.79E-22	0.20583712	0.09906706	1.04E-06	0.01452512	0.05702061	0.6904386
1.93E-46	0.37920633	0.00549562	0.38150796	0.26099047	0.24506809	0.97675211
0.66045261	2.91E-18	1.22E-57	5.75E-08	4.66E-11	0.31692976	0.60894006
0.43900353	7.76E-40	5.34E-05	2.66E-05	6.98E-06	0.00094702	0.84377601
0.66556542	1.46E-119	5.83E-09	0.84550258	0.0029224	0.00909001	0.77072519
0.18167184	0.64513246	0.01856295	0.12870262	0.02735295	0.06652559	0.14577181
0.03879347	2.10E-12	3.94E-19	3.75E-10	0.00139559	4.52E-09	0.00059847
0.76637755	0.19756581	0.54805063	0.34780801	0.74344742	0.00227031	0
0.06066845	9.47E-29	2.24E-41	0.21743431	3.79E-15	3.86E-07	1.87E-07
0.49802051	1.14E-06	1.24E-10	0.15510055	5.39E-10	0.00022315	0.45903133
0.46671556	3.10E-11	0.00078157	0.11379981	0.34962099	0.05431146	0.85236809
0.2897242	7.44E-99	2.73E-36	0.24451249	2.71E-08	2.01E-05	0.62895678
0.78404359	0.5918366	0.40811271	1.98E-17	1.37E-14	0.3653117	0.458779
0.00113307	2.41E-33	0.00037871	0.08221324	1.46E-07	3.11E-08	0.90592668
0.83515438	2.02E-05	0.00061288	0.11187095	0.46861938	0.47038462	1.87E-09
0.9504382	2.28E-80	0.05616249	4.44E-06	0.34962819	0.00644601	0.55806313
1.43E-11	0.03413401	5.76E-14	4.62E-09	0.00410398	0.78319497	0.43326803
0.78395992	0.00284318	0.34340455	3.71E-13	0.0041509	0.00046794	0.25835247
0.32628525	6.12E-13	1.61E-40	0.74360226	0.84335049	3.74E-16	0.05202652
6.20E-22	0.4257871	0.36631519	3.22E-76	0.04094971	0.6035802	0.80541743
0.25412534	6.20E-39	1.42E-11	0.04301665	2.07E-06	1.22E-14	0.55122702

CPT1C	PPARD	APOC3	RXRG	UCP1	ACSL4	EHHADH
0.01364262	4.16E-13	0.27427761	0.02328419	0.01070604	3.23E-24	0.00933033
0.81871484	0.55062707	0.91584845	0.3715872	0.6619359	0.90211834	0.12093489
6.57E-08	1.94E-06	0.00269656	0.17277439	0.24718379	9.94E-16	1.00E-56
0.00056621	0.20705303	0.67107799	0.00012438	3.75E-05	0.05459916	0.60758864
1.50E-08	1.02E-05	0.00025601	0.03134379	0.52168869	4.71E-16	2.78E-55
0.02640205	0.81868422	0.33473181	0.41470581	0.02466742	4.40E-12	8.85E-19
0.00431217	0.98595827	0.05171825	0.01393586	0.04637136	1.78E-08	4.56E-16
0.07100254	1.82E-08	0.51482878	0.04549558	0.73074464	0.5634169	0.35361761
1.57E-06	0.37881575	0.33893797	0.00534261	0.12995979	0.80345286	0.05655378
2.39E-05	0.00052095	2.57E-45	0.40065247	0.3973914	2.53E-06	8.04E-11
9.50E-26	2.70E-05	0.46702054	0.00440726	0.79193737	8.35E-06	6.53E-14
0.60154559	0.04823732	0.9539087	0.54101608	0.00183177	8.18E-11	0.12209739
0.22121159	0.00454914	0.68592166	0.00067217	0.00185759	7.90E-06	5.51E-23
0.06249926	0.84387716	0.24296713	0.14279617	0.31529514	0.64490877	0.00637502
0.68470317	0.00577474	0.6540555	6.23E-06	0.95018784	0.81015181	2.72E-05
0.01293738	1.83E-07	0.51221864	0.03213478	0.0427451	4.13E-21	5.24E-05
0.29886363	0.89249464	0.80094124	0.71987016	0.08952895	1.19E-24	0.16328003
0.89732922	0.05624208	0.85519703	0.43818979	0.98404007	0.10942889	0.08721499
1.13E-05	3.71E-08	0.14653379	0.08396069	0.89977015	0.02888067	0.12600687
6.67E-07	1.12E-05	1.06E-23	0.33782385	0.36761414	8.32E-18	5.89E-29
5.85E-13	3.10E-06	0.20907609	0.67585658	0.54564322	1.85E-12	8.53E-32
6.66E-17	2.81E-11	0.72850752	0.06192917	0.27670325	6.66E-16	3.80E-69
0.61940878	0.04337983	0	0.40057474	0.51613831	0.76150591	0.50562648
0.30877079	0.01472773	0.51252979	2.90E-05	0.05375374	2.49E-09	0.00013905
1.56E-06	3.07E-15	0.08394507	0.00019936	0.19653554	0.00879859	0.80972086
0.39715678	0.22749694	0	0.93843745	0.94956667	0.61800648	0.86308944
0.80763916	0.2238874	0.64989397	4.82E-16	9.45E-33	0.95690578	0.91621691
0.39085557	0.17290712	0	0.04087218	0.9224135	0.61389782	0.99302654
2.74E-14	4.27E-08	0.2799987	0.04654143	0.2294814	1.49E-11	1.20E-51
0.55219647	0.66175585	0.59485492	0.2166623	0.26925151	2.03E-05	0.04347875
0.21130436	2.64E-09	0.12390567	0.00129541	0.00796453	3.57E-19	0.27980649
3.31E-08	0.03202603	0.77381072	0.00397974	0.0203697	0.00019238	1.96E-101
0.01593069	0.31408306	0.61938229	0.0238798	0.78851151	1.45E-07	0.73756982
6.78E-09	2.76E-05	0.78054612	4.26E-05	0.32446453	2.09E-05	1.80E-18
0.0453087	0.15026061	0.94736242	0.2901659	4.36E-08	0.31252555	0.03794743
0.20246469	1.78E-07	0.49921966	0.26169099	0.76078121	2.02E-17	0.10347081
1.78E-05	0.04833781	0.79303913	0.00056319	0.03489938	0.01109386	2.63E-80
0.02051395	0.08149919	0.72036511	0.00071484	0.78549249	0.46323771	2.97E-07
0.0328468	0.66282075	0.88619057	1.57E-22	1.00E-42	0.2836137	0.17164379
5.03E-15	1.08E-11	2.12E-05	0.13054111	0.93092094	7.55E-27	4.56E-79
0.00861894	1.02E-05	0.8710473	0.00026934	0.5043675	5.90E-10	5.71E-10
0.00333715	0.28451785	0.78010169	4.79E-22	1.93E-46	0.66045261	0.43900353
2.40E-10	2.54E-06	0.18488578	0.20583712	0.37920633	2.91E-18	7.76E-40
0.0028364	4.47E-12	0.52978783	0.09906706	0.00549562	1.22E-57	5.34E-05
0.00031307	8.01E-08	0.3449973	1.04E-06	0.38150796	5.75E-08	2.66E-05
0.13914303	0.10607343	0.7351112	0.01452512	0.26099047	4.66E-11	6.98E-06

3.73E-10	1.48E-05	0.00285516	0.05702061	0.24506809	0.31692976	0.00094702
0.3838213	0.22946352	0	0.6904386	0.97675211	0.60894006	0.84377601
0	9.07E-09	0.3400458	0.00070822	1.23E-06	1.13E-06	1.10E-15
9.07E-09	0	0.19623262	0.72841217	0.92464338	8.48E-07	0.00259113
0.3400458	0.19623262	0	0.9602207	0.99359835	0.64884079	0.94051744
0.00070822	0.72841217	0.9602207	0	1.75E-22	0.59545717	0.35801175
1.23E-06	0.92464338	0.99359835	1.75E-22	0	0.62699983	0.00817841
1.13E-06	8.48E-07	0.64884079	0.59545717	0.62699983	0	0.00099994
1.10E-15	0.00259113	0.94051744	0.35801175	0.00817841	0.00099994	0
1.05E-06	0.00052033	0.61279888	0.04366861	0.14030647	1.33E-15	1.92E-55
2.24E-07	0.16982594	0.11034814	0.06038095	0.60039347	0.00039498	0.00267298
0.00036856	6.40E-05	0.00038848	0.01625363	0.00030586	0.00018265	0.35599377
0.34613776	0.19918493	0	0.80108473	0.99320247	0.67227347	0.90570977
7.33E-20	3.35E-11	1.59E-07	0.17191133	0.27869257	2.19E-30	1.51E-51
0.00669952	0.00955767	0.54399047	0.01772673	0.03104753	4.82E-14	0.00297851
0.00032148	0.00558446	0.94196525	0.96474753	0.02603139	7.92E-08	6.61E-07
1.97E-17	1.95E-10	0.68249264	0.061987	0.48716682	3.16E-26	3.37E-82
7.81E-06	0.90479858	0.36334045	0.00012182	0.42122283	7.53E-09	1.30E-25
4.08E-06	0.00013065	0.82952318	0.00014524	0.00225388	0.55233291	7.82E-25
3.13E-11	0.49963929	2.65E-09	0.46705444	0.95430233	0.00014542	0.07178767
0.00025663	0.10921506	0.52136652	0.0008384	0.06734673	0.00192245	3.38E-51
0.2917245	0.05898089	0.37125207	1.63E-05	0.00358377	0.0144911	0.22524238
0.00191869	3.44E-05	0.17627384	0.00340139	0.37440951	0.02209989	5.16E-07
4.98E-17	3.46E-14	0.05361206	0.13421813	0.07570555	0.00025605	2.08E-16
0.15608821	0.05656929	0.76725734	8.05E-16	9.59E-05	0.06201935	0.0624559
5.96E-17	2.80E-06	0.57557518	0.00416053	0.20259131	0.00196596	7.27E-88

PCK1	RXRA	PLIN5	APOA1	SCP2	SLC27A1	FABP2
1.05E-08	0.23481043	4.62E-12	0.28533277	2.46E-14	0.00820352	0.00011166
0.32727523	0.13874796	0.85088092	0.91346471	0.22775966	0.36496548	0.97783367
6.10E-143	0.00745659	2.57E-07	0.00318334	6.40E-29	4.08E-05	5.69E-12
0.50318088	8.86E-05	6.94E-11	0.71902565	0.03744441	0.00384946	0.46624645
2.13E-105	0.00177079	8.20E-09	0.00027945	1.08E-27	0.05202637	7.48E-16
0.1211286	0.00478724	1.63E-14	0.34125433	0.29126998	0.79206413	0.02033764
3.01E-05	0.55985938	0.00021594	0.05050194	0.1132237	0.11511688	0.83768264
0.0017499	0.02932799	0.0098008	0.47594514	0.8768577	0.75627168	0.57888869
0.07699909	4.32E-08	0.00023181	0.3504761	0.9812173	0.19930004	0.27690455
3.19E-16	0.21222507	4.65E-07	3.66E-45	3.03E-14	0.00890778	8.90E-06
1.09E-06	0.28205571	3.52E-08	0.50999608	1.66E-11	0.47883219	0.00074518
0.70969741	0.34136071	0.12635234	0.99659037	0.03056056	0.27888648	0.69336408
1.70E-49	0.52545452	2.49E-08	0.66242913	2.80E-07	0.01470355	1.03E-16
0.39098092	0.21940496	0.44307598	0.23458793	0.03814051	0.31380614	0.60200746
0.00329177	0.89239297	0.27738259	0.70736038	0.00263349	0.88384665	0.07538257
2.65E-13	0.00932084	8.45E-14	0.53031541	3.07E-15	0.00069617	2.05E-07
0.00011986	5.28E-05	9.10E-07	0.75296321	2.05E-14	2.03E-05	0.02097879
2.31E-08	0.94070061	9.23E-05	0.81876202	0.67080895	0.00186821	0.38494234
0.05999998	0.00040652	1.62E-05	0.171284	5.65E-05	0.50073108	0.03588499
4.38E-98	0.04344953	3.63E-11	9.79E-24	5.69E-29	4.83E-06	1.12E-08
5.85E-10	0.01761481	1.03E-06	0.20624497	1.06E-30	0.0580195	2.58E-05
4.97E-85	0.66123256	3.29E-10	0.74452721	9.11E-38	4.54E-07	2.92E-12
0.40563739	0.18321387	1.23E-06	0	1.85E-06	0.11544131	0.89823334
3.14E-06	0.83283056	0.18143129	0.50797099	2.05E-07	0.42686123	0.47867909
0.83467885	0.05035982	0.55640107	0.0935186	8.38E-06	1.45E-06	0.0926249
0.7306385	0.13019233	0.00050546	0	2.82E-07	0.46332634	0.84742874
0.85348101	0.10945883	9.19E-16	0.70315114	0.25678124	0.00071208	0.04677083
0.73600257	0.24863531	0.00080575	0	3.09E-08	0.32884956	0.99998223
1.89E-47	0.21755246	2.18E-19	0.32407684	1.97E-33	0.0098909	8.75E-15
0.00178967	0.71128147	0.00029055	0.56474694	0.02526309	0.75062981	0.11909481
8.62E-08	0.20720529	1.20E-44	0.12405431	0.00110067	0.5840193	0.08788994
4.87E-75	0.02078309	0.81233853	0.77539538	1.63E-32	0.03920039	2.82E-12
5.70E-08	0.02778377	2.47E-06	0.59293857	0.02299934	0.59666416	0.02473598
2.85E-13	0.39434261	3.28E-15	0.75300248	4.99E-18	0.65522172	0.14790961
0.38938071	0.97336331	0.0129569	0.96590077	0.97600642	0.61390108	0.99840515
4.37E-05	0.65299505	0.24379829	0.50012785	3.63E-07	0.38774627	0.06508711
1.68E-74	0.19697193	0.37978728	0.79244136	1.98E-20	0.01604851	9.51E-08
0.0010553	0.44959286	0.423222	0.61632264	5.09E-05	0.01117679	0.81976151
0.86511289	0.16507919	0.00817448	0.8804951	0.58170797	0.76198955	0.3020531
6.44E-83	0.46285453	2.69E-09	2.61E-05	1.20E-66	4.70E-14	1.53E-18
8.41E-14	0.10053962	0.00173997	0.88515994	3.69E-11	0.0825047	0.00020039
0.66556542	0.18167184	0.03879347	0.76637755	0.06066845	0.49802051	0.46671556
1.46E-119	0.64513246	2.10E-12	0.19756581	9.47E-29	1.14E-06	3.10E-11
5.83E-09	0.01856295	3.94E-19	0.54805063	2.24E-41	1.24E-10	0.00078157
0.84550258	0.12870262	3.75E-10	0.34780801	0.21743431	0.15510055	0.11379981
0.0029224	0.02735295	0.00139559	0.74344742	3.79E-15	5.39E-10	0.34962099

0.00909001	0.06652559	4.52E-09	0.00227031	3.86E-07	0.00022315	0.05431146
0.77072519	0.14577181	0.00059847	0	1.87E-07	0.45903133	0.85236809
1.05E-06	2.24E-07	0.00036856	0.34613776	7.33E-20	0.00669952	0.00032148
0.00052033	0.16982594	6.40E-05	0.19918493	3.35E-11	0.00955767	0.00558446
0.61279888	0.11034814	0.00038848	0	1.59E-07	0.54399047	0.94196525
0.04366861	0.06038095	0.01625363	0.80108473	0.17191133	0.01772673	0.96474753
0.14030647	0.60039347	0.00030586	0.99320247	0.27869257	0.03104753	0.02603139
1.33E-15	0.00039498	0.00018265	0.67227347	2.19E-30	4.82E-14	7.92E-08
1.92E-55	0.00267298	0.35599377	0.90570977	1.51E-51	0.00297851	6.61E-07
0	0.0008239	2.00E-08	0.64803491	3.10E-26	2.81E-05	5.97E-11
0.0008239	0	0.00190041	0.13909847	0.11511461	1.54E-09	0.49274858
2.00E-08	0.00190041	0	0.00045997	0.00526837	0.91596599	0.00653352
0.64803491	0.13909847	0.00045997	0	1.51E-07	0.49628624	0.91994599
3.10E-26	0.11511461	0.00526837	1.51E-07	0	6.60E-13	7.68E-07
2.81E-05	1.54E-09	0.91596599	0.49628624	6.60E-13	0	0.0022104
5.97E-11	0.49274858	0.00653352	0.91994599	7.68E-07	0.0022104	0
4.97E-70	0.57169569	3.94E-06	0.67621534	4.06E-98	6.71E-12	1.08E-07
0.11497377	0.0480267	0.10101455	0.38113217	1.58E-05	0.27351321	0.29781882
2.30E-26	0.86564649	1.62E-14	0.87168766	2.54E-09	0.53030942	0.00012052
0.00553439	0.00272841	0.00110883	2.12E-09	0.08846309	0.34161026	0.0141523
1.48E-68	0.34150561	0.11798884	0.51101689	1.69E-16	0.0133049	0.00790294
0.00235419	0.05357315	0.000518	0.41045043	4.19E-06	0.00182041	0.23936171
0.13445586	3.24E-06	1.45E-12	0.18986068	0.25616747	0.00997441	0.00033218
7.29E-10	0.09565133	3.30E-29	0.05418147	4.02E-25	0.9199475	0.12916115
0.01051257	0.64762079	0.60983578	0.77951807	0.47245321	0.0253056	0.87765544
1.00E-28	0.45429739	0.00804571	0.57137373	5.78E-64	0.00174554	0.00029235

ACADM	ACADL	AQP7	FADS2	CYP4A22	LPL	ILK
5.22E-21	0.00304683	0.00017666	2.94E-08	0.23802345	3.50E-06	0.16535241
0.09609275	0.13683898	0.22949797	0.75629238	0.39097326	0.53773249	0.06732621
2.47E-88	0.60404603	4.29E-28	0.00966949	9.75E-64	0.10666657	0.02350262
0.21502922	3.68E-15	7.99E-09	8.06E-05	0.08564634	0.00014645	0.09395746
2.20E-82	0.70040279	1.62E-24	0.00822932	5.84E-48	0.04339921	0.20566982
0.90810379	4.21E-26	0.00027813	0.00053941	1.18E-05	0.00461845	1.38E-12
0.05651329	4.13E-23	4.98E-06	0.59313395	5.12E-12	0.39085254	8.31E-05
0.00936543	0.03962348	1.83E-07	0.00028549	6.33E-05	0.19201276	1.48E-07
0.58970543	0.17985333	0.00242623	0.9707995	0.2049095	5.84E-06	0.49069869
3.12E-11	0.00454825	0.17701016	7.69E-05	1.17E-08	0.00686795	0.00080166
1.36E-16	8.47E-07	1.70E-07	0.00079416	0.00018263	0.94308132	0.52795301
0.93239896	0.30665334	0.55154937	0.57059196	0.50407728	0.54359215	0.03702257
3.41E-40	0.20305819	2.55E-16	0.68911111	3.71E-27	0.2447912	1.26E-08
0.15762683	0.79010219	0.60669154	0.61835413	0.46163185	0.2487516	0.04146152
0.00762646	8.95E-14	4.63E-05	0.42775401	4.65E-05	0.16134448	0.07862302
1.17E-22	0.05507123	7.64E-08	1.53E-06	0.00029964	7.00E-05	0.02079552
4.44E-11	0.05700517	0.02235664	0.00013892	0.07841555	4.49E-07	0.0337601
0.00081067	0.03502193	4.85E-11	0.41537755	1.12E-06	0.00041128	0.04653961
0.00047317	0.00771504	2.97E-06	0.33127744	0.48230476	0.03239809	1.79E-06
7.46E-70	0.01001269	3.70E-19	0.28049081	4.42E-60	0.28179524	8.17E-05
3.33E-43	3.14E-08	6.29E-07	0.00120721	0.00239085	2.41E-06	3.29E-07
7.97E-98	6.03E-10	2.81E-36	0.00013973	2.72E-50	0.1763148	0.08442371
0.82560091	0.23976524	0.38286909	1.30E-08	0.42842882	0.41495199	0.03697946
2.81E-09	0.02608528	0.00015031	0.01904179	0.00035133	3.57E-09	5.45E-12
0.98249536	8.25E-07	4.55E-07	0.0022873	0.05214541	0.00131312	6.40E-19
0.59153996	0.36208018	0.89179944	1.64E-09	0.58755696	0.39452626	0.21605792
0.6930634	0.39054115	1.39E-08	0.83362118	0.71903758	2.81E-08	0.17696505
0.96267309	0.908893	0.92709044	2.69E-09	0.32135968	0.40399145	0.38205684
3.81E-83	2.91E-08	3.65E-31	0.00065587	4.88E-22	4.44E-13	0.13907172
0.00038466	0.38264765	0.0007762	0.0056548	0.07989953	0.0198344	0.16324094
8.91E-09	2.04E-05	6.00E-11	0.02498253	0.02649195	6.53E-05	3.48E-07
3.61E-65	1.63E-16	1.33E-26	0.00162269	2.46E-48	0.06919152	4.11E-06
3.31E-07	0.53217932	5.48E-07	1.81E-12	1.04E-05	0.00307273	0.34884769
1.98E-37	2.79E-19	6.45E-23	0.0035612	4.42E-15	1.38E-11	2.03E-05
0.64682834	0.64820058	0.26272239	0.70896275	0.14785179	0.16879633	0.10862581
5.81E-14	5.65E-05	0.55169382	2.55E-05	0.10010149	3.76E-07	0.33389657
1.03E-49	8.86E-16	6.49E-40	0.28676857	9.63E-207	0.13834661	0.07465758
4.52E-09	9.92E-18	1.75E-07	0.08553424	3.57E-05	6.08E-06	1.00E-06
0.95387703	0.19506998	0.00102064	0.73950164	0.82949221	1.40E-16	0.89093454
2.83E-118	0.00531059	1.37E-25	0.00852913	1.38E-43	1.80E-06	0.83500526
1.02E-18	0.46656503	1.83E-06	0.04523083	3.74E-08	6.46E-06	0.15765331
0.2897242	0.78404359	0.00113307	0.83515438	0.9504382	1.43E-11	0.78395992
7.44E-99	0.5918366	2.41E-33	2.02E-05	2.28E-80	0.03413401	0.00284318
2.73E-36	0.40811271	0.00037871	0.00061288	0.05616249	5.76E-14	0.34340455
0.24451249	1.98E-17	0.08221324	0.11187095	4.44E-06	4.62E-09	3.71E-13
2.71E-08	1.37E-14	1.46E-07	0.46861938	0.34962819	0.00410398	0.0041509

2.01E-05	0.3653117	3.11E-08	0.47038462	0.00644601	0.78319497	0.00046794
0.62895678	0.458779	0.90592668	1.87E-09	0.55806313	0.43326803	0.25835247
1.97E-17	7.81E-06	4.08E-06	3.13E-11	0.00025663	0.2917245	0.00191869
1.95E-10	0.90479858	0.00013065	0.49963929	0.10921506	0.05898089	3.44E-05
0.68249264	0.36334045	0.82952318	2.65E-09	0.52136652	0.37125207	0.17627384
0.061987	0.00012182	0.00014524	0.46705444	0.0008384	1.63E-05	0.00340139
0.48716682	0.42122283	0.00225388	0.95430233	0.06734673	0.00358377	0.37440951
3.16E-26	7.53E-09	0.55233291	0.00014542	0.00192245	0.0144911	0.02209989
3.37E-82	1.30E-25	7.82E-25	0.07178767	3.38E-51	0.22524238	5.16E-07
4.97E-70	0.11497377	2.30E-26	0.00553439	1.48E-68	0.00235419	0.13445586
0.57169569	0.0480267	0.86564649	0.00272841	0.34150561	0.05357315	3.24E-06
3.94E-06	0.10101455	1.62E-14	0.00110883	0.11798884	0.000518	1.45E-12
0.67621534	0.38113217	0.87168766	2.12E-09	0.51101689	0.41045043	0.18986068
4.06E-98	1.58E-05	2.54E-09	0.08846309	1.69E-16	4.19E-06	0.25616747
6.71E-12	0.27351321	0.53030942	0.34161026	0.0133049	0.00182041	0.00997441
1.08E-07	0.29781882	0.00012052	0.0141523	0.00790294	0.23936171	0.00033218
0	2.35E-05	1.55E-24	3.41E-05	1.67E-45	6.49E-10	0.0817342
2.35E-05	0	5.55E-13	0.75584211	8.61E-10	0.96918983	3.46E-06
1.55E-24	5.55E-13	0	0.00013468	1.59E-35	6.53E-05	0.44013462
3.41E-05	0.75584211	0.00013468	0	0.23773909	0.04590679	0.00159871
1.67E-45	8.61E-10	1.59E-35	0.23773909	0	0.05551608	0.69957954
6.49E-10	0.96918983	6.53E-05	0.04590679	0.05551608	0	0.09280683
0.0817342	3.46E-06	0.44013462	0.00159871	0.69957954	0.09280683	0
6.37E-33	3.87E-09	2.74E-27	0.01071182	8.47E-11	3.97E-11	0.19179265
0.96263732	0.00090356	7.54E-08	0.66879281	1.11E-09	1.15E-07	0.00164871
2.51E-92	1.19E-13	1.47E-27	0.00629405	2.19E-36	8.90E-05	1.21E-08

ACSL1	FABP4	CPT2
2.83E-09	0.06856707	1.63E-06
0.52767467	0.64703443	0.05863876
5.09E-08	0.64983119	4.91E-28
3.29E-30	1.86E-13	0.03594612
6.71E-11	0.78232414	8.63E-28
0.00022517	0.00492704	3.01E-12
0.40084071	0.00189732	1.98E-09
0.00875871	0.93067484	0.00127636
0.0011366	0.04166542	0.637174
0.29341525	0.03408952	0.00060502
9.22E-13	0.27362281	1.80E-18
0.2931377	0.7789546	0.66765586
0.05673742	0.44223424	5.88E-09
0.56414486	0.33555513	0.00883153
0.63241026	0.00017889	0.35858604
4.26E-10	0.12956882	7.56E-09
1.22E-09	0.07880842	0.00014237
3.24E-06	0.02871273	0.04275506
2.92E-06	0.07862899	7.49E-05
1.72E-07	0.50232988	1.46E-17
3.81E-32	0.14515661	6.04E-37
5.03E-20	0.57548055	1.29E-36
0.02666474	0.7415279	0.43584654
6.25E-09	0.00687625	6.69E-06
0.9905767	0.00769133	0.08882505
0.05339895	0.79570575	0.52779445
2.73E-05	9.23E-13	0.00552962
0.01992422	0.72894022	0.89094356
4.86E-54	0.94883495	9.13E-52
6.97E-06	0.10048858	0.00568899
1.10E-26	0.53670741	3.08E-07
9.90E-09	0.00044074	4.20E-53
0.01560384	0.91814012	0.00092934
2.52E-77	0.0131741	2.11E-45
0.00107906	0.45937198	0.90698674
0.61254231	0.32673002	0.0001448
3.00E-11	2.36E-08	2.89E-48
3.38E-06	0.00478394	7.30E-08
0.6549296	1.88E-23	0.57493451
7.60E-30	0.77968862	8.41E-64
2.87E-08	0.11418395	3.92E-13
0.32628525	6.20E-22	0.25412534
6.12E-13	0.4257871	6.20E-39
1.61E-40	0.36631519	1.42E-11
0.74360226	3.22E-76	0.04301665
0.84335049	0.04094971	2.07E-06

3.74E-16	0.6035802	1.22E-14
0.05202652	0.80541743	0.55122702
4.98E-17	0.15608821	5.96E-17
3.46E-14	0.05656929	2.80E-06
0.05361206	0.76725734	0.57557518
0.13421813	8.05E-16	0.00416053
0.07570555	9.59E-05	0.20259131
0.00025605	0.06201935	0.00196596
2.08E-16	0.0624559	7.27E-88
7.29E-10	0.01051257	1.00E-28
0.09565133	0.64762079	0.45429739
3.30E-29	0.60983578	0.00804571
0.05418147	0.77951807	0.57137373
4.02E-25	0.47245321	5.78E-64
0.9199475	0.0253056	0.00174554
0.12916115	0.87765544	0.00029235
6.37E-33	0.96263732	2.51E-92
3.87E-09	0.00090356	1.19E-13
2.74E-27	7.54E-08	1.47E-27
0.01071182	0.66879281	0.00629405
8.47E-11	1.11E-09	2.19E-36
3.97E-11	1.15E-07	8.90E-05
0.19179265	0.00164871	1.21E-08
0	0.07969157	1.71E-40
0.07969157	0	0.00405648
1.71E-40	0.00405648	0
